# Twenty two cases of canine neural angiostrongylosis in eastern Australia (2002-2005) and a review of the literature

**DOI:** 10.1186/1756-3305-5-70

**Published:** 2012-04-05

**Authors:** Julian A Lunn, Rogan Lee, Joanna Smaller, Bruce M MacKay, Terry King, Geraldine B Hunt, Patricia Martin, Mark B Krockenberger, Derek Spielman, Richard Malik

**Affiliations:** 1 Faculty of Veterinary Science, The University of Sydney, Sydney, NSW 2006 Australia; 2 ICMPR, Westmead Clinical School, Westmead Hospital, Westmead, NSW 2065 Australia; 3 Veterinary Specialist Services, Corner Lexington & Logan Roads, Underwod, QLD 4119 Australia; 4 Centre for Veterinary Education, The University of Sydney, B22, Sydney, NSW 2006 Australia

**Keywords:** Ivermectin, Peripheral Eosinophilia, Moxidectin, Contemporary Cohort, Crude Antigen

## Abstract

Cases of canine neural angiostrongylosis (NA) with cerebrospinal fluid (CSF) evaluations in the peer-reviewed literature were tabulated. All cases were from Australia. A retrospective cohort of 59 dogs was contrasted with a series of 22 new cases where NA was diagnosed by the presence of both eosinophilic pleocytosis and anti-*Angiostrongylus cantonensis* immunloglobulins (IgG) in CSF, determined by ELISA or Western blot. Both cohorts were drawn from south east Queensland and Sydney. The retrospective cohort comprised mostly pups presented for hind limb weakness with hyperaesthesia, a mixture of upper motor neurone (UMN) and lower motor neurone (LMN) signs in the hind limbs and urinary incontinence. Signs were attributed to larval migration through peripheral nerves, nerve roots, spinal cord and brain associated with an ascending eosinophilic meningo-encephomyelitis. The contemporary cohort consisted of a mixture of pups, young adult and mature dogs, with a wider range of signs including (i) paraparesis/proprioceptive ataxia (ii) lumbar and tail base hyperaesthesia, (iii) multi-focal central nervous system dysfunction, or (iv) focal disease with neck pain, cranial neuropathy and altered mentation. Cases were seen throughout the year, most between April and July (inclusive). There was a preponderance of large breeds. Often littermates, or multiple animals from the same kennel, were affected simultaneously or sequentially. A presumptive diagnosis was based on consistent signs, proximity to rats, ingestion/chewing of slugs or snails and eosinophilic pleocytosis. NA was diagnosed by demonstrating anti-*A. cantonensis* IgG in CSF. Detecting anti-*A. cantonensis* IgG in serum was unhelpful because many normal dogs (20/21 pound dogs; 8/22 of a hospital population) had such antibodies, often at substantial titres. Most NA cases in the contemporary series (19/22) and many pups (16/38) in the retrospective cohort were managed successfully using high doses of prednisolone and opioids. Treatment often included antibiotics administered in case protozoan encephalomyelitis or translocated bacterial meningitis was present. Supportive measures included bladder care and physiotherapy. Several dogs were left with permanent neural deficits. Dogs are an important sentinel species for NA. Human cases and numerous cases in tawny frogmouths were reported from the same regions as affected dogs over the study period.

**Electronic supplementary material:**

The online version of this article (doi:10.1186/1756-3305-5-70) contains supplementary material, which is available to authorized users.

## Background

*Angiostrongylus cantonensis* is a metastrongyloid nematode that normally lives in the right ventricle and pulmonary arteries of rats, its definitive (permissive) host [1]. While many species of rats can carry patent infections, the Norwegian rat (*Rattus norvegicus*) and the black rat (*Rattus rattus*) are considered the most important definitive hosts. In wild populations of rats, *A. cantonensis* infections cause little disease, as expected for an efficient parasite [1–3]. Dogs, humans, horses, Australian native mammals (e.g. possums, macropods, macrobats) and birds (e.g. tawny frogmouths), and various zoo animals are non-permissive "accidental" hosts that become infected after ingesting third-stage larvae (L_3_) in intermediate hosts (molluscs) [1, 4] or transport hosts (such as planarians, frogs, fish and crustaceans) [5–7]. Tawny frogmouths and Australian marsupials are highly susceptible to clinical neural angiostrongylosis.

In rats, following digestion, L_3_ migrate from the gut to peripheral nerves, nerve roots, spinal cord and brain [8]. *A. cantonensis* shows obligate neurotropism, i.e. larvae must migrate through the central nervous system (CNS) before taking up residence in the pulmonary arteries, where they subsequently mate and produce eggs which embolise in the pulmonary capillary bed. Larvae migrate up the trachea, then are coughed up, swallowed and passed in the stool, where they access intermediate mollusc hosts (slugs or snails). Virtually all species of native and introduced terrestrial molluscs in Australia are suitable intermediate hosts [1].

Larval neurotropism dominates disease pathogenesis in non-permissive hosts like dogs and people. In human patients, signs of NA include headache, fever, nausea, vomiting, neck stiffness, paraesthesia, face or limb paralysis, photophobia, diplopia, coma, seizures and even death [9–11]. Canine NA usually results from ingestion of slugs, snails or paratenic hosts containing infective L_3_[12–15]. After ingestion, larvae leave the gut, typically via intestinal veins and lymphatics. They then travel up peripheral nerves and nerve roots, subsequently moving cranially within the spinal cord parenchyma or via the subarachnoid space. They damage CNS tissues via two mechanisms. Firstly, there is neural injury and haemorrhage (mechanical damage and cavitations) consequent to the migration process [16, 17]. Additionally, intense eosinophilic meningo-encephalomyelitis is triggered by larval antigens, most likely metabolic, excretory or moulting products [18–20]. Thus, combinations of pathomechanisms injure the CNS in various locations and through different processes, resulting in a range of neurologic signs. The use of anthelmintics (e.g. levamisole, benzimidazoles, avermectins) cause larvae to die, which ends the mechanical damage but greatly increase parasite-derived eosinophilic inflammation, due to sudden release of cytoplasmic metazoan antigens and cessation of production of parasite-derived immunosuppressant molecules.

Three elements are required for infection to occur: (i) the presence of rats, (ii) the presence of a suitable intermediate mollusc hosts and (iii) the opportunity for the potential host to eat (or chew) infected definitive, intermediate or paratenic hosts. In Australia, the disease in dogs (especially pups) was well known in south east Queensland as a result of seminal studies by Ken Mason and colleagues in the 1970s [21, 22]. As a result, cases are commonly diagnosed and treated in general practice. Although some of this work is published, the actual Master's dissertation [14] contains additional pertinent information. Subsequently, the disease was reported in an increasing variety of native and domestic species, initially in Queensland and later in Sydney. The expansion of disease was likely attributable to extension in the geographic range of some critical mollusc host. In the early 1990s, disease in pet dogs was reported for the first time in Sydney, and subsequently has been seen increasingly as a life-threatening cause of neurologic disease in dogs, horses, macropods, possums, macrobats, birds (tawny frogmouths) and animals in zoological collections (especially monkeys) throughout its geographic range along eastern Australia. It has likewise become a cause of life-threatening neurologic disease in humans over the same range, especially in infants (who have a propensity to eat slugs and snails) [11] and inebriated adult males (typically inebriated young men who eat a snail for a dare, typically at a "buck's night").

In 2003, two of the authors (JL & RM) encountered the disease in a young dog [23]. This provided the impetus to utilise a relatively specific serological test, thus permitting more definitive diagnosis of NA based on detecting Ig directed against *A. cantonensis* rather than making a presumptive diagnosis based on consistent signs and an eosinophilic pleocytosis. The further refinement and validation of ELISA and Western blot assays for NA permitted the authors to re-examine the range of clinical findings in dogs with eosinophilic meningoencepalomyelitis/encephalitis, to determine whether there was a wider spectrum of neurologic sequelae to infection with *A. cantonensis*.

This article has two components: a review of the canine literature and a new series of cases collected prospectively with the help of colleagues in practice and veterinary pathologists. The findings of this contemporary study are examined in relation to the human and experimental literature concerning NA.

## Methods

### A. Literature review of documented cases

Relevant papers concerning NA in dogs were identified by searching Commonwealth Agricultural Bureau (CAB) and OVID Medline databases using the key words "cantonensis" and "dog or dogs or canine". Data concerning clinical findings, CSF cytology, necropsy observations and serology were collated. Only cases with CSF analysis were included in the study cohort. In the largest recorded case series of NA, a grading scale was used to divide patients into groups based on the severity of clinical signs [14]. Grade 1 cases had hind limb involvement and were mildly affected, Grade 2 cases progressed to involve the forelimbs and occasionally the cranial nerves, while Grade 3 cases progressed rapidly to severe generalised paralysis and hyperaesthesia, and were euthanased on humane grounds.

### B. Contemporary case series

#### Case recruitment

Hospital records and clinicopathologic data from dogs with suspected NA were recruited from practices in the vicinity of Sydney and Brisbane between January 2001 and May 2005. A letter was sent to all registered Small Animal Specialists and Veterinary Pathologists in Queensland and New South Wales outlining the study aims and samples desired from suspect NA cases (Additional file 1: Appendix 1). After contact with the primary clinician, a questionnaire (Additional file 2: Appendix 2) was sent to determine the onset of the signs, disease features, clinical course, administration of parasiticides and exposure to intermediate hosts. Data including signalment, presenting complaints, clinical pathology (including necropsy findings) and treatment outcomes were collected. CSF and/or serum specimens were obtained, where possible, for serological studies. Samples were stored at -80°C prior to analysis.

#### Inclusion criteria

Case inclusion was typically based on a presumptive diagnosis of NA, i.e. cases with progressive neurologic signs, a history of ingesting rats, snugs or snails, proximity to rats and eosinophilic pleocytosis in CSF. A more definitive diagnosis was based on identifying nematode larvae within the CNS at necropsy or detecting specific Ig against *A. cantonensis* in CSF. Of 30 dogs considered initially, only 22 had CSF cytology data (and thus CSF available for ELISA and/or Western blot testing for anti-*A. cantonensis* Ig). These 22 dogs were recruited into the study. No cases in this prospective group were confirmed at necropsy. Dogs with an atypical presentation were included only if they were positive for Ig against *A. cantonensis*. Six dogs (a litter of greyhound pups) had strong epidemiologic support for a diagnosis of NA, including positive necropsy in one pup and were only included as a footnote. CSF findings were unavailable for two presumptive cases, which were therefore not included.

#### Treatment

All dogs were treated with analgesic and supportive care, as required, plus glucocorticoids (dexamethasone [initially in some cases] and/or prednisolone). Supportive care included opioids, intravenous fluid therapy, urinary catheterisation or manual expression of the bladder, antimicrobials and passive physiotherapy. Pred-nisolone dosage ranged from 0.5 to 2 mg/kg (divided daily); the dose was subsequently tapered over an extended period, typically 6-12 weeks. A minimum of 4 weeks glucocorticoid therapy was given in all instances. No dogs were given anthelmintics as part of the treatment regimen, although some had been administered prior to diagnosis as part of routine heartworm/flea prophylaxis.

### C. Detection of anti-*A. Cantonensis* antibodies

#### ELISA methods

Serum and CSF from suspected cases of NA were tested using an ELISA developed by one of the authors (RL). Positive and negative controls were tested also. IgG against *A. cantonensis* was detected by an indirect ELISA, adapted from the method of Cross & Chen [24]. Methods for obtaining antigen are outlined in Additional file 3: Appendix 3. The technique used to perform the ELISA is described briefly, as follows. The extract of adult *A. cantonensis* (crude antigen) was coated onto Maxisorb plates at a concentration of 5 μg/mL. Serum and CSF were diluted to 1:100 in "Blotto". Two-fold, serial dilutions of 1:100 serum or CSF were made. Dilutions from 1:100 to 1:204,800 were tested in each assay. The secondary antibody, rabbit anti-dog IgG, conjugated to horseradish peroxidase (HRP), was added to each well at a concentration of 1:1,000. Primary and secondary incubation steps were carried out at 37°C for 1 h, with washes in distilled water between steps. Substrate was allowed to react for 5 min at room temperature and then stopped with 1 M phosphoric acid. Wells were read at 450 nm using a Tecan plate reader.

#### ELISA cut-off values

**Figure 1:**
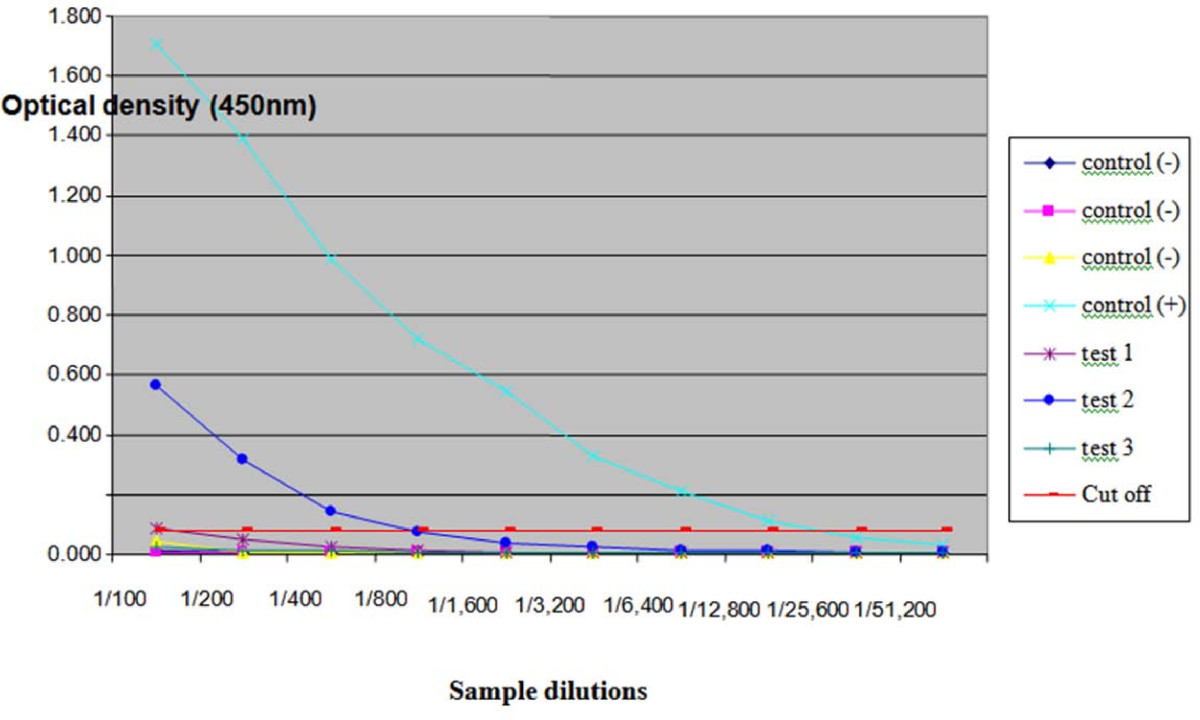
**Calculation of ELISA cut-off value**. The optical density (OD) of three negative control sera was determined at serum dilutions of 1:100 to 1:512,000. E.g., for the dilution of 1:100: negative control 1 = 0.011, negative control 2 = 0.008, negative control 3 = 0.044. The mean (***x***) and standard deviation (SD) of these three negative sera were calculated, viz. ***x***= 0.021, SD = 0.02. The cut-off value was taken as ***x***+ 3SD, in this example 0.083. Thus, an OD value above this value was considered positive. The antibody titres for test samples 1 and 2 were determined to be 1:100 and 1:800, respectively (i.e. where the line interpolated between the dots crosses the **red** cut-off threshold), but the titre for sample 3 was zero. Thus, test sample 1 and 2 are considered positive.

**Figure 2:**
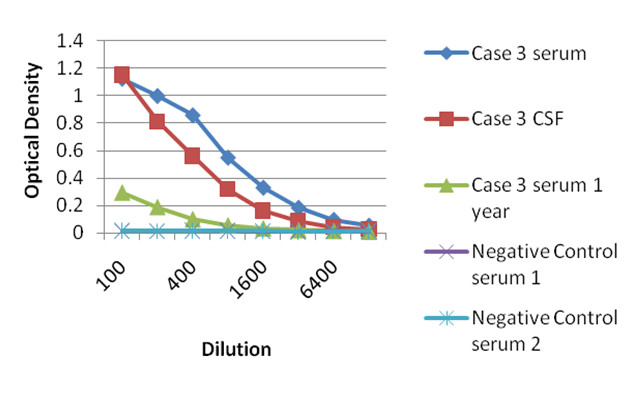
**ELISA results for serum and CSF from case 3 of our series**. Note the optical density in the serum sample is still positive one year after therapy, but much lower than at the time of presentation.

The titre of serially diluted serum or CSF was calculated by comparison with the optical density (OD) of three known negative controls (mature adult pound dogs that were clinically normal in all respects). Serum and CSF from a necropsy-confirmed case of NA were used as positive controls. Validation of the ELISA using control specimens is illustrated in Figures 1 and 2. The OD of 1 to 3 negative sera and 1 negative CSF specimen were determined at a dilution of 1:100 for each batch of specimens tested. The same positive and negative controls were used for each batch of specimens assayed. The mean of the control serum or CSF samples, plus three standard deviations (SD) was considered the cut-off value above which the next dilution of the unknown serum or CSF was considered positive (Figure 1). Based on the results, using the cut-off values as described above, descriptive statistics were calculated.

#### Western blot analysis

Many serum and CSF specimens were also tested for *A. cantonensis* antibodies using an assay developed by one of the authors (JS) as a component of a project concerning NA in macropods [25]. Detection of IgG to adult *A. cantonensis* antigens was carried out on specimens using Western blot analyses. The technique has been described in detail elsewhere [25]. Briefly, polyacrylamide gel electrophoresis in sodium dodecyl sulphate (SDS-PAGE) was used to separate antigens of an adult *A. cantonensis* preparation. The electrophoresed proteins were transferred to a nitrocellulose membrane via a semi-dry technique [26]. The antigen-infused nitrocellulose membrane was incubated overnight at room temperature with the test specimen. Following a final wash, the membrane was incubated with a secondary antibody, rabbit anti-dog IgG conjugated to HRP. A chromagen solution was then added.

Based on human serological studies, the 31 kDa and the 204 kDa antigens were used to determine the presence or absence of IgG to *A. cantonensis*[27–31]. The molecular weight (MW) of the observed bands was determined for each nitrocellulose strip by comparison with the migration of known MW markers. CSF specimens were considered positive if there were either discrete bands or wider strips of colour at an apparent MW of 31 or 204 kDa. The serum antibody response was measured subjectively by visualising the density of the band and graded as either negative (-), weakly positive (+), positive (2+) or strongly positive (3+). For purposes of this study, all positive responses were grouped together.

#### Control serum and CSF specimens

Samples of CSF and/or serum were collected from two groups of dogs for use as negative controls. Samples were obtained from 21 dogs (Group A) euthanased at pounds during September 2005. The age of these dogs was unknown but based on physical appearance and dentition, they were probably less than one-year-old. All were cross-breds. There were 14 entire males and 7 females of unknown reproductive status. Immediately after euthanasia, blood was collected by percutaneous cardiac puncture and CSF by cisternal tap. Serum was harvested from heart blood. A small section of the spinal cord was obtained from a region adjacent to the cisterna magna. The presence or absence of inflammation within cord sections was determined histologically. A second group of controls (Group B) consisted of client-owned dogs presenting for reasons unrelated to possible infection with *A. cantonensis*. Serum was collected from dogs undergoing routine desexing or orthopaedic procedures during May 2005. None of these cases had neurologic dysfunction. Twenty-two dogs of mixed ages and breeds provided serum samples. There were 15 males and 7 females; 10 of the males were castrated and four of the females were spayed. The median age of the group was 4 years (range: 5 months to 12 years), i.e. older than Group A dogs. All Group B dogs were on heartworm (*Dirofilaria immitis*) prophylaxis and were regularly treated with anthelmintics for intestinal parasites. Details of Control Group B are summarised in Additional file 4: Appendix 4.

## Results

### A. Cases recorded previously

#### Signalment

**Figure 3:**
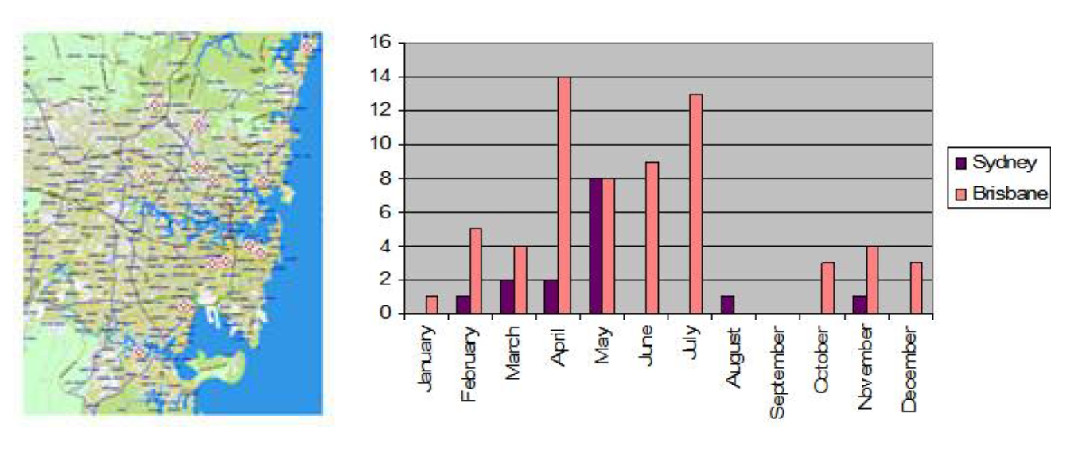
**Geographic and temporal distribution of prospective cases in the Sydney region**. Individual cases are indicated by a red dot on the map on the left, and in purple columns (y axis - number of cases; x axis - month of the year) in the schematic on the right.

The literature contained descriptions of 59 dogs with naturally-occurring NA based on CSF cytology, clinical findings or necropsy data [13–15, 32]. Published case details are summarised in Additional file 5: Appendix 5. Of the 59 cases, only 38 met the inclusion criteria (i.e. availability of CSF data). Thirty-seven were published by Mason [14] from south-eastern Queensland, with only one by Collins *et al.*[32] from Sydney.^A^ The median age of this retrospective cohort was 10 weeks (range 6 to 28 weeks). The month in which cases were seen is shown in Figure 3 and Additional file 6: Appendix 6; cases tended to present between April and July. Of the 38 cases, 23 had siblings affected (i.e. two or more members of a litter affected simultaneously or sequentially). Breeds represented are listed in Table 1. Of the 38 cases, 31 (82%) were large or giant breed dogs and 14 (37%) were racing greyhounds. There were 23 males and 15 females, none of which were desexed.

**Table 1:** Signalment and CSF cytology in 38 cases of NA from the literature (retrospective cohort)

Case No.	Breed	Age (weeks)	Gender	Month	CSF Protein (g/L)	CSF Nucleated cell count (cells/μL)	CSF Eosinophils		Necropsy confirmed (NA larvae present)
							**Number (cells/μL)**	**% nucleated cells**	
***Mason*** ^***a***^									
Case 2	Border Collie	9	M	October	0.12	4200	3696	88%	?
Case 3*	Daschund	14	M	January	N/A	7125	6056	85%	No
Case 13*	Kelpie	6	F	December	N/A	3680	2760	75%	No
Case 14*	Kelpie	7	M	December	N/A	1300	1053	81%	No
Case 15*	Labrador	8	F	April	N/A	2307	2030	88%	No
Case 16*	Greyhound	10	F	July	0.43	4600	3404	74%	Yes
Case 17*	Greyhound	8	M	July	N/A	2700	1782	66%	Yes
Case 18*	Greyhound	10	M	July	1.62	7750	6743	87%	Yes
Case 19*	Greyhound	9	M	July	0.13	284	244	86%	No
Case 20*	Greyhound	10	F	May	N/A	265	98	37%	No
Case 21*	Corgi	9	F	April	2.3	1350	1053	78%	No
Case 22*	Labrador	7	M	December	N/A	90	55	61%	No
Case 27*	GSD	7	F	February	N/A	1250	1025	82%	Yes
Case 28*	GSD	7	M	February	0.2	2264	1449	64%	No
Case 29*	Greyhound	8	M	April	0.25	2812	2418	86%	No
Case 31*	Greyhound	10	F	June	0.32	1280	1139	89%	No
Case 32	Greyhound	11	F	June	0.18	3250	2633	81%	?
Case 33	Greyhound	11	M	June	0.19	1465	1231	84%	?
Case 34	Greyhound	11	F	June	0.23	240	226	94%	?
Case 35	Labrador	11	M	April	N/A	20	20	100%	?
Case 36	Afghan	11	M	June	0.43	2300	1702	74%	?
Case 37	Beagle	10	M	May	0.12	83	59	71%	?
Case 38*	OESD	9	F	July	0.4	2376	1925	81%	Yes
Case 39	OESD	10	M	July	0.11	7106	6680	94%	?
Case 40	OESD	9	F	July	0.12	1197	1029	86%	?
Case 41	OESD	9	M	July	0.12	2510	2309	92%	?
Case 42	Borzoi	8	F	November	N/A	512	435	85%	?
Case 43*	Daschund	12	M	October	N/A	2800	1652	59%	Yes
Case 44	Greyhound	8	M	July	0.92	700	462	66%	?
Case 45*	Greyhound	10	M	May	N/A	1760	1390	79%	Yes
Case 46*	Greyhound	9	M	July	2.1	450	95	21%	Yes
Case 49	Labrador	12	F	November	N/A	406	256	63%	?
Case 50	Great Dane	20	F	April	0.9	1590	350	22%	?
Case 51	Great Dane	28	M	February	0.6	2750	2063	75%	?
Case 52	Poodle	14	M	May	2.4	250	150	60%	?
Case 53*	Weimaraner	12	F	April	0.5	490	441	90%	?
Case 54*	Greyhound	16	M	May	N/A	2880	2304	80%	Yes
**Collins** ***et al.*** * ^**b**^	Bull Terrier	9	M	May	0.54	886	886	100%	Yes
	**Median**	**10**		**June**			**1185**	**81%**	

OESD - Old English Sheep dog

GSD - German Shepherd dog

*Died or euthanased

N/A - not available

?- not known

^a^Mason [14]; ^b^Collins *et al.* [32]

#### History and clinical presentation

All cases had bilateral hind limb paresis, hind limb muscle wasting, urinary bladder paresis/incontinence, tail paresis and hyperaesthesia. Less common findings included gastrointestinal signs (vomiting, diarrhoea) and various neurologic deficits (cranial nerve palsies, faecal incontinence, convulsions or coma).

#### Clinical pathology

**Figure 4:**
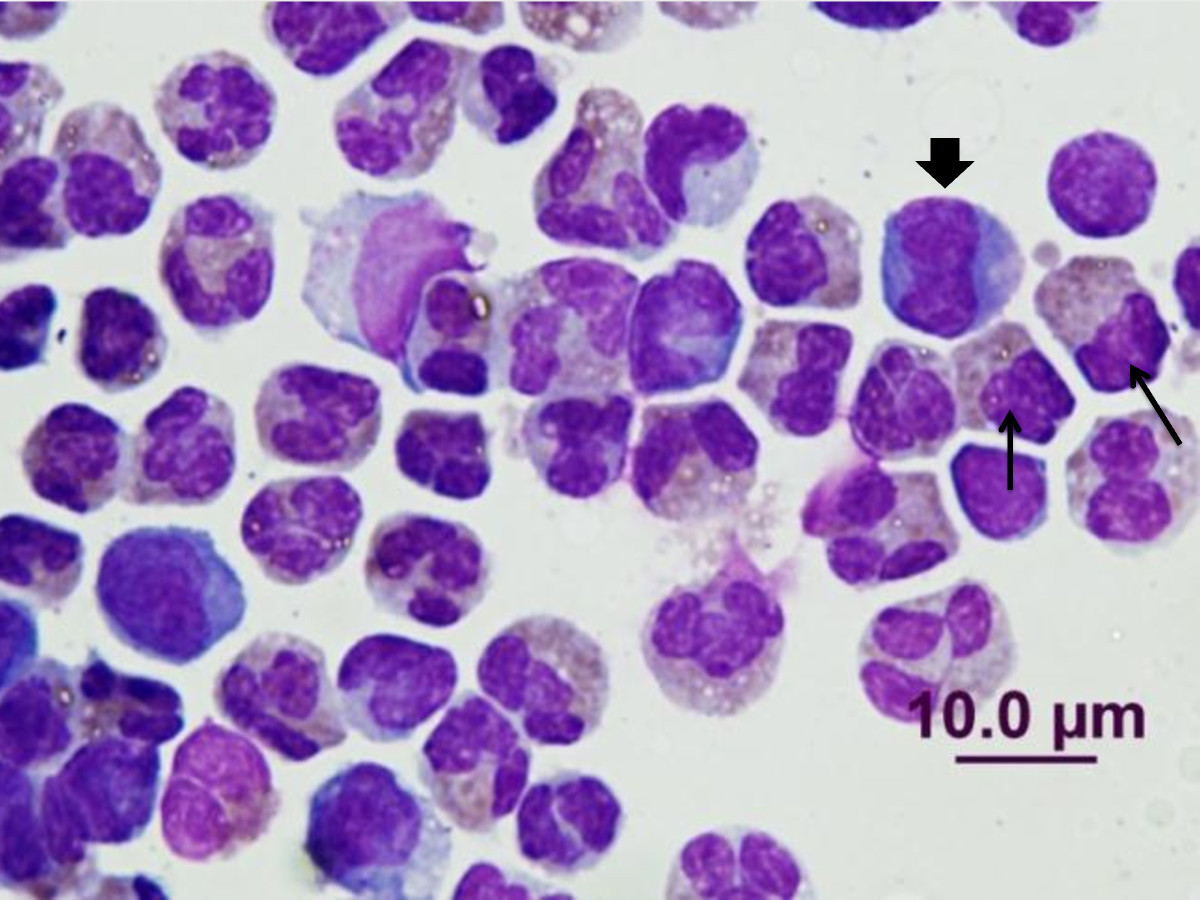
**Cytospin smear of CSF specimen from Case 2 (contemporary series) stained with DiffQuik showing eosinophilic pleocytosis**. Two eosinophils are marked with a black arrow, although many more are present in the smear. A macrophage is marked with an arrowhead. Segmented neutrophils are present also.

Haematology was performed in most instances. The only consistent finding was peripheral eosinophilia. CSF demonstrated an eosinophilic pleocytosis (Figure 4; Table 2). The median eosinophil count within CSF was 1,185 cells/μL (range: 20 to 6,743 cells/μL). The median percentage of eosinophils was 81% (range: 22% to 100%).

**Table 2:** Signalment and CSF cytology of contemporary cases of NA

						CSF Data		
**Case No**.	**Breed**	**Age (weeks)**	**Gender**	**Month**	**Protein (g/L)**	**Nucleated Cells (× 10** ^**6**^ **/L)**	**Eosinophils**	
							**cells/μL**	**%**
1	Labrador	24	M	May	0.3	270	232	86%
2	Golden Retriever	20	F	May	0.5	4660	3,821	82%
3	Kelpie	24	F	May	0.28	182	173	95%
4	Golden Retriever	16	F	March	0.55	458	412	90%
5	Boxer	16	F	May	0.55	458	389	85%
6	Miniature Poodle	16	M	May	1.35	332	282	85%
7	Cross Bred	16	M	June	1	146	145	99%
8	Beagle	20	F	July	0.61	3651	3,505	96%
9	Australian Cattle Dog	48	F	February	0.55	5952	4,940	83%
10	Staffordshire Terrier	8	F	February	1.07	1,800	1,134	63%
11	Labrador	32	F	April	1	6,970	5,576	80%
12	Labrador	192	F	August	0.86	1782	1,550	87%
13*	Boxer	48	M	May	0.28	182.2	173	95%
14	Kelpie	96	F	July	1	4683	4,449	95%
15	Staffordshire Bull Terrier	400	F	November	1.28	6825	6,279	92%
16	Cross Bred	192	F	April	0.11	4.5	0.23	5%
17	Maltese	96	M	March	0.4	1746	1,397	80%
18	Dobermann	44	F	June	2.82	6.2	1	13%
19*	German Shorthaired-Pointer	288	M	March	3.3	7860	4,716	60%
20	Rhodesian Ridgeback	32	F	March	0.31	351	260	74%
21	Cross Bred	10	M	May	0.86	132	125	95%
22*	English Bulldog	16	M	November	0.23	15	14	95%
	**Median**	**28**					**401**	**86%**

* Cases 13, 19 and 22 were euthanased at their owner's request.

#### Treatment outcomes

All dogs received supportive care viz. manual expression of the urinary bladder and passive range of motion exercises. Many cases received empiric antibiotic therapy (details not available). Betamethasone (1 to 2 mg/kg) was administered subcutaneously or orally for 3 to 7 days. The mortality rate was 58% (22/38 dogs not surviving). The initial severity of the clinical signs did not appear strongly associated with outcome; 9 of the 20 dogs (45%) classified as Grade 1 were euthanased, 5 of the 11 (45%) Grade 2 dogs were euthanased or died, while all of the Grade 3 cases were euthanased. Only one dog received anthelmintics (Case 3; Grade 3) but it was euthanased soon after treatment commenced. Of the remaining 16 cases that survived, at least 3 cases (Cases 2, 50 and 51) had permanent neurologic deficits.

### B. Contemporary study

#### Signalment

Samples of serum and or CSF from 22 dogs with presumptive NA were collected between January 2002 and May 2005. There were 8 males (4 castrated) and 14 females (7 spayed). No breed predisposition was detected, although 13 (59%) of the dogs were large breeds. Specifically, there were 3 cross breds, 3 Labrador retrievers, 2 each of Boxers, Kelpies, Golden Retrievers and Staffordshire Bull Terriers, and one each of Maltese, Cattle Dog, Beagle, British Bulldog, Dobermann, German Shorthair Pointer, Miniature Poodle and Rhodesian Ridgeback. The median age of cases was 28 weeks (range: 8 weeks to 10 years). None of the cases had litter mates affected.^B^ Most (64%) of cases were presented between April and July (Figure 3; Additional file 4: Appendix 4). Figure 3 shows the geographic distribution of cases in the Sydney metropolitan area, suggesting that the parasite is not concentrated in particular suburbs.

#### History and presenting complaints

Only one dog (Case 5) was observed eating slugs and snails. The route of infection for the remaining 21 cases was not identified, although several clients mentioned rats in their immediate area. The most common signs at presentation were hyperaesthesia (14 cases), posterior proprioceptive ataxia (10), hind limb weakness (7), cranial nerve dysfunction (e.g. lack of a menace response, facial twitching or ocular signs) (7), urinary incontinence (6), UMN hyperreflexia (5), altered mentation (4) and muscle atrophy (1). These results are summarised in Table 2. History and physical findings for each case are described in detail in Additional file 7: Appendix 7. Two dogs received avermectins for heartworm prophylaxis in the two weeks prior to signs of NA emerging (Cases 2 and 20), although signs were probably unrelated in time to dosing in these instances.

#### Clinical pathology

Of the 22 dogs, 21 had eosinophilic pleocytosis in CSF (Figure 4); the remaining dog had an eosinophil count of 1 cell/μL, with 10% of the count comprising eosinophils. A diagnosis of eosinophilic pleocytosis in humans requires greater than 10% eosinophils of a total CSF cell count, and also greater than 10 eosinophils/μL, [7, 33] and this definition has been adopted for dogs. Samples were obtained from the cisterna magna in all dogs except Cases 16 and 20, which had CSF collected via lumbar puncture immediately before a caudal myelogram. The median eosinophils count of CSF was 401 cells/μL, while the median percentage eosinophils was 86%. CSF results are summarised in Table 2. Haematology was performed concurrently in 10/22 dogs, of which eight (80%) demonstrated peripheral eosinophilia. No significant biochemical abnormalities were noted within the study cohort.

#### Diagnostic imaging

Myelograms performed in Cases 16 and 20 did not reveal abnormalities apart from diffuse spinal cord swelling in both instances and rapid clearance of iohexol from the subarachnoid space in Case 20. Case 18 had computed tomography (CT) of the head and neck (before and after intravenous iohexol), with no abnormalities detected.

#### Treatment outcomes

On the whole, the prognosis was good to excellent, with death or euthanasia in only 3/22 cases (14%). The length of prednisolone administration varied from 4 weeks to 3 months. All but four dogs (Cases 13, 18, 19 & 22) improved rapidly over the initial 48 h and went onto make a complete recovery. Some dogs received antibiotics prior to administration of glucocorticoids, usually with a view to "covering" the alternative diagnosis of protozoan myelopathy (neosporosis or toxoplasmosis); accordingly, clindamycin, trimethoprim-sulpha and pyrimethamine were the most commonly chosen drugs. Two dogs (Cases 13 and 22) were euthanased due to ongoing, unresponsive behavioural problems. Case 13 improved at first but deteriorated over the subsequent 4 months and was euthanased due to inappropriate aggression. Case 22 was treated for 4 weeks with no improvement and euthanasia was elected because of aggression. Case 19 had a two weeks history of anisocoria, cervical pain and altered behaviour; cervical pain resolved with glucocorticoids but ocular signs progressed to blindness and the dog was euthanased. Unfortunately none of these dogs were examined at necropsy following euthanasia. Case 18 presented with head tremors that proved unresponsive to treatment; its quality of life was considered adequate and the owners declined further investigation or treatment.

### ELISA data

#### Controls

Of the 21 dogs in Group A (young pound dogs), 20 (95%) had a measurable titre of anti-*A. cantonensis* IgG in serum (Table 3). There was, however, no demonstrable anti-*A. cantonensis* IgG in CSF specimens in 20/21 dogs. The single dog (Control A15) with an antibody titre in CSF of 100 had the highest serum titre (6,400). This specimen, along with specimens from Control A8 and Control A13, were contaminated with blood during collection and this likely accounted for the low CSF antibody titre detected. Within Group B, 8/22 dogs (36%) had measurable serum anti-*A. cantonensis* IgG (Table 3); CSF was not available from these individuals. The antibody titres in control group A (young pound dogs) were substantially higher than titres in the older hospital patients.

**Table 3:** ELISA for control dogs i.e. dogs without NA

Control Group A			Control Group B	
**Case**	**Serum**	**CSF**	**Case**	**Serum**
1	1:800	Negative	1	1:800
2	1:400	Negative	2	Negative
3	1:800	Negative	3	1:100
4	1:800	Negative	4	1:100
5	1:200	Negative	5	Negative
6	1:1,600	Negative	6	Negative
7	1:1,600	Negative	7	1:100
8	1:200	Negative	8	Negative
9	1:800	Negative	9	1:100
10	1:800	Negative	10	Negative
11	Negative	Negative	11	Negative
12	1:200	Negative	12	Negative
13	1:100	Negative	13	Negative
14	1:800	Negative	14	1:200
15	1:6,400	1:100	15	Negative
16	1:100	Negative	16	1:400
17	1:800	Negative	17	Negative
18	1:800	Negative	18	Negative
19	1:100	Negative	19	Negative
20	1:400	Negative	20	Negative
21	1:400	Negative	21	Negative
			22	1:100

Group A dogs were young pound dogs, while Group B were clinical patients in a veterinary hospital

#### Contemporary NA cases

**Figure 5:**
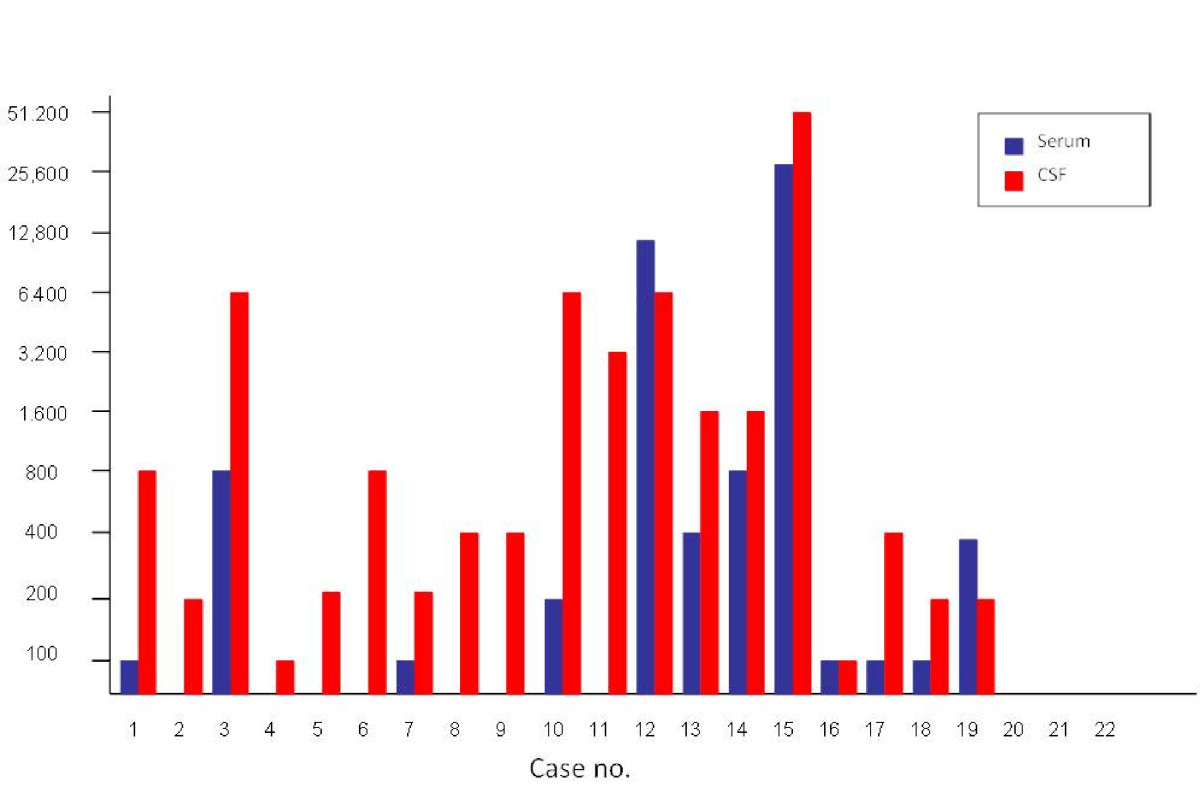
**Antibody titres in serum and CSF samples from the retrospective/prospective study cohort**. The vertical axis represents the reciprocal titre of antibodies against *A. cantonensis*. Serum titres are shown in blue, whereas CSF titres are shown in red. Cases 21 and 22 had no titre detected using ELISA, but were positive using Western blot analysis for the 31 kDa antigen.

Of cases where CSF was available for testing, 19/21 (90%) were positive for anti-*A. cantonensis* IgG using the ELISA. Of these 19 cases, serum was available for 16, of which 12 were positive also using the ELISA. Thus, four cases with negative serum titres had positive IgG titres in CSF. In eight patients, the titre in CSF exceeded that in serum, whereas in two cases the opposite was true, while in one further patient the titres in serum and CSF were equal. Two patients (Cases 1 and 3) had serum samples tested one year after the initial sample (Figure 2). Both were positive, with titres of 1:100 and 1:200, compared to 1:100 and 1:800 at first admission. Both cases that were negative for CSF antibodies using ELISA were also negative for serum antibodies. These results are summarised in Table 3 and Figure 5.

#### Western blot data

Fifteen cases had serum and or CSF submitted for Western Blot assay and all reacted to the 31 KDa antigen band, including one case that was negative on CSF and serum (Case 20). None of the dogs demonstrated antibodies to the 204 kDa antigen. The results are summarised in Table 4.

**Table 4:** ELISA and Western Blot assay results for the Contemporary Group

Case	ELISA (actual titre)		ELISA (positive ***v*** negative)		Western Blot
	**Serum**	**CSF**	**Serum**	**CSF**	
1	1:100	1:800	Positive	Positive	Positive
2	Negative	1:200	Negative	Positive	Positive
3	1:800	1:6,400	Positive	Positive	Positive
4	Negative	1:100	Negative	Positive	Positive
5	N/A	1:200	N/A	Positive	N/A
6	Negative	1:800	Negative	Positive	Positive
7	1:100	1:200	Positive	Positive	Positive
8	Negative	1:400	Negative	Positive	Positive
9	N/A	1:400	N/A	Positive	Positive
10	1:200	1:6,400	Positive	Positive	N/A
11	N/A	1:3,200	N/A	Positive	N/A
12	1:12,800	1:6,400	Positive	Positive	Positive
13	1:400	1:1,600	Positive	Positive	N/A
14	1:800	1:1,600	Positive	Positive	Positive
15	1:25,600	1:51,200	Positive	Positive	N/A
16	1:100	1:100	Positive	Positive	Positive
17	1:100	1:400	Positive	Positive	Positive
18	1:100	1:200	Positive	Positive	Positive
19	1:400	1:200	Positive	Positive	N/A
20	Negative	Negative	Negative	Positive	Positive
21	Negative	N/A	Negative	N/A	Positive
22	Negative	Negative	Negative	Negative	N/A
**Positive (%)**	**63%**	**90%**	**63%**	**90%**	**100%**

## Discussion

### Retrospective case series

One aim of this study was to compare cases published previously to those seen prospectively. It is clear that there are a number of differences between the two study cohorts. When Mason *et al.* published the first series of NA cases in 1976 [21], he described a characteristic syndrome affecting pups (less than 20-weeks-old) consisting of hind limb paresis, lumbar hyperaesthesia and urinary incontinence. Therefore, the entire cohort of 55 cases published in 1983 [14] and the five dogs documented by Collins *et al.*[32] were presumptively diagnosed as having NA based on a combination of almost pathognomonic physical findings. The diagnosis was then substantiated by CSF cytology and/or necropsy. Many patients initially exhibited only one or two of the three signs. Furthermore, many dogs went onto develop additional signs (e.g. forelimb paresis, cranial nerve palsies, altered mentation, coma, seizures) although these were not included as part of the classic "syndrome". Thus, the retrospective group could be considered to have clinical signs of an ascending meningo-encephalomyelopathy, with conspicuous involvement of the lumbosacral nerve roots.

In most respects, signs in young puppies mirror those observed in children, especially infants, perhaps reflecting an immune system inexperienced at dealing with metazoan parasites, a large synchronous infective dose of L_3_ combined with a small vertebral canal, spinal cord and intervertebral foraminae. In adult humans, NA tends be self-limiting with mild clinical signs [9], although there are exceptions. The most common presenting complaints include headache (98%), neck pain (70%), fever (30%) and vomiting (35%) [10]. Cranial nerve signs and spinal neuropathy are seen mainly in children [11], associated with a higher mortality and increased frequency of permanent neurologic sequelae [9]. Fatalities although rare, do occur, especially in children [11, 34, 35]. There may also be signs associated with ingestion and migration of the larvae such as fever, malaise, vomiting and diarrhoea [36].

### Contemporary case series

In comparison to the historical cohort, most dogs in the contemporary arm of this study did not demonstrate Mason's triad of signs [14]. However, they all demonstrated clinical signs consistent with a progressive meningo-encephalomyelopathy and many presentations were more reminiscent of the range of signs and symptoms seen in adult human patients. Consequently, we extended our conceptual framework to include NA as a potential cause of neurologic disease in dogs of any age with progressive meningo-encephalomyelopathy, eosinophilic pleocytosis and the presence of anti-*A. cantonensis* IgG in CSF.

### Morbidity and mortality

The recorded cases of NA in pups had a combined mortality rate of 59%, compared to 14% for our contemporary group. The three cases that were euthanased in the prospective cohort were thought to have had permanent neurologic deficits and two of these (Cases 13 and 22) were young. Permanent sequelae are often observed in human patients with NA, often due to the effects of granulomas or abscesses that form around dead and dying larvae or worms in the CNS parenchyma or peripheral (typically cranial) nerves [16, 34]. Mason does not give a figure for the percentage of surviving puppies demonstrating permanent neurologic deficits, but mentions it as a common occurrence, with at least one case euthanased as a result.

### Pathophysiology and differential diagnosis

Signs of progressive meningo-encephalomyelopathy are seen with a number of other diseases, including distemper, neosporosis, toxoplasmosis, cryptococcosis and other fungal diseases; indeed, the list of conditions is much longer in countries outside Australia [20, 37–44]. There are some clinical features, however, which assist in differentiating NA from other diseases. First, large breed dogs appear over represented, presumably because they have a greater opportunity to ingest molluscs in a predominately outdoor domicile. Secondly, there tend to be a mixture of LMN and UMN signs, the balance changing with disease progression, with a tendency for the major "neuroanatomic lesion" to ascend during the clinical course. Thirdly, hyperaesthesia is the most consistent and conspicuous presenting sign. In our series, the location and extent of hyperaesthesia was more varied than in cases recorded to date, with cervical pain more prominent in many cases than lumbar or caudal hyperaesthesia. In Mason's series, all cases had posterior hyperaesthesia, whereas in ours 16/23 dogs (70%) displayed hyperaesthesia, but only six with lumbar involvement. Eleven dogs (50%) presented with cervical pain/nuchal rigidity and in four of these (Cases 1, 8, 11 and 16) this was the only presenting sign. This is reminiscent of the picture in adult humans, where the most common symptoms are headache and neck pain [9].

Hyperaesthesia likely reflects peripheral radiculoneuritis and eosinophilic meningitis. Large areas of eosinophilic infiltration are associated with spinal nerve roots in experimentally- and naturally-infected dogs [13, 14]. Hyperaesthesia may also be associated with migration of L_3_ along peripheral nerves, particularly the sciatic nerve; this has been documented in experimentally infected dogs, rabbits and mice [13, 17, 45]. *Pneumostrongylus tenius* larvae invade the CNS of Canadian White-tailed deer via peripheral nerves, especially those in the lumbar muscles [46]. This migration pattern may occur with *A. cantonensis* in dogs and would account for lumbar pain being a common early sign. Such a neuropathic pathomechanism suggests the potential use of gabapentin or pregabalin as adjunctive drugs for managing pain in such cases.

Two factors seem critical in determining clinical features in a given species: i) the initial infective dose, and ii) the host's innate and acquired ability to prevent larvae reaching the CNS. In many non-permissive hosts, larvae fail to reach the CNS because an effective and timely immune response prevents their migration. Thus, guinea pigs, calves, pigs and rhesus monkeys can withstand dosing with 1000's of larvae without developing signs [47–49]. The host's nutrition status, concurrent and previous parasite burdens and stress potentially affect the ability of *A. cantonensis* larvae to migrate from gut to CNS. Mason documented numerous young puppies with concurrent intestinal parasitism [14]. Such pre-existing infections could compromise mucosal integrity, facilitating penetration of the gut by L_3_. Interestingly, a number of our cases had a stressful episode prior to the onset of clinical disease (e.g. surgery, chemotherapy, immunosuppressive therapy) that may have adversely affected immune defence mechanisms.

The initial penetration of the gut by L_3_ is accompanied by vomiting or abdominal pain in many species [13, 15, 16] and Mason noted that vomiting often preceded neurologic signs by 7-10 days. Antecedent alimentary signs were noted in some of our patients. Vomiting may also occur when neurologic signs develop. The "incubation period" for NA was generally indeterminable. In experimental infections, signs develop 9-14 days post-inoculation, although the parasite can be seen in the CNS within three days of ingestion [13, 14]. In humans, the incubation period ranges from 1-36 days, with an average of 12-16 days [16, 50–52]. This period coincides with the second moult (from L_4_ to L_5_ stage young adults), a time associated with a rise in circulating immune complexes, enlargement of neural granulomas and enhanced meningeal inflammation in experimentally infected animals [53, 54].

### Epidemiology

There is a trend within both study cohorts for cases to present from mid-autumn to early winter (April to July). This has also been observed in human cases occurring in Brisbane [55]. Although dogs are more likely to develop NA during this period, cases can present in any month. Evaluating the two region's climates, peak incidence coincides with periods of high humidity and when the average daily temperature ranges from 17°-25°C (Additional file: Appendix 6). The rate of larval development within intermediate hosts depends on ambient temperature, with an ideal range of 21°-26°C [1]. Once larvae have matured to the L_3_ stage, they become quiescent and remain viable for the life of the mollusc. In other regions, seasonal peaks in human NA relate to key intermediate or paratenic host populations. In Tahiti, cases are most common when water prawns are abundant [56]; while in New Caledonia, it is when native molluscs are most numerous [57]. In Thailand, there is no seasonal peak because small aquatic snails (*Pila sp*.), the usual source of infection, are abundant throughout the year [9].

In dogs, the most likely route of infection is direct ingestion of an intermediate host. While only one case in our series (Case 5) was actually seen to eat slugs and snails, Mason noted that many of his cases had ingested molluscs. In Australia, a number of species of slugs and snails can serve as intermediate hosts. These may contain as many as 1000 infective larvae [35]. Larvae remain viable within a dead intermediate host for as long as 11 days [57]. Mason showed experimentally that pups would eat slugs even when other food was offered and suggested that the normal "mouthing" behaviour of puppies might allow ingestion of larvae [14]. Such behaviours are less likely to be exhibited by adult dogs, thus contamination of food may be more important in these cases. Infective larvae are secreted within the mucus trails of molluscs and they can survive for several hours therein, depending on ambient environment [58].

### Diagnosis

Definitive diagnosis of NA has historically been made at necropsy, or by identifying larvae of appropriate morphology within CSF or aqueous humour samples. In the future, it might be possible to detect cavitations caused by migrating L_3_ using high field magnetic resonance imaging (MRI), as is done in human patients [12], or using a recently developed PCR assay that detects *A. cantonensis* DNA in blood or CSF (see later)[59].

Ten of the previously recorded canine NA cases were confirmed at necropsy, but none had larvae detected in CSF specimens. Collection of CSF is easier in humans than dogs, as there is no requirement for anaesthesia. Furthermore much larger volumes are obtained and in several countries repeated lumbar puncture is considered part of therapy for NA [9, 16]. In a series of human cases, 54% of patients had elevated CSF opening pressures [9]. Increased CSF pressure contributes to clinical signs, notably headache and neck pain, consequently, patients may have CSF drained more than once. Patients, including children, will routinely have up to 25 mL of CSF removed [60, 61]. Accordingly, the chance of recovering larvae is greater than in dogs where typically 2 mL of CSF or less is obtained. Most veterinarians are hesitant in removing large volumes of CSF from any patient, due to a perceived risk of the cerebellar herniation. In Australia, larvae have yet to be identified in the CSF of human NA patients antemortem, which may also reflect our physicians' caution or lower numbers of larvae in CSF [62].

As most dogs in the contemporary cohort recovered, diagnosis of NA was based on consistent signs, eosinophilic pleocytosis in CSF and response to therapy. In all cases except one (Case 22), the presence of specific IgG against *A. cantonensis* provided additional supportive evidence. The percentage eosinophils and absolute eosinophil counts in both study cohorts were similar, with most dogs having both high percentages (> 76%) and high cell counts (> 1,600 cells/μL). Indeed, in the retrospective cohort, all dogs had eosinophilic pleocytosis. In the contemporary cohort, however, two dogs (Cases 16 and 18) had < 10 eosinophils/μL. Case 16 displayed signs consistent with progressive meningoencephalo-myelitis and responded to glucocorticoids, while neurologic signs in Case 18 persisted despite treatment; both dogs were positive for *A. cantonensis* IgG in serum and CSF using ELISA and Western blots. Therefore, despite these dogs not having met the strict criteria for eosinophilic pleocytosis, they were still considered to have NA. Similar conclusions were made in a recent outbreak of human NA in Taiwan, with only 12/17 cases (71%) developing eosinophilic pleocytosis [61]. Interestingly, of these 12 cases, only 5 initially had eosinophilic pleocytosis; the remaining 7 developed it over the subsequent 10 days (despite some patients receiving glucocorticoids). Thus, lack of eosinophilic pleocytosis should not rule out NA. Indeed, based on experimental observations, some animals with NA are expected to not demonstrate eosinophilic pleocytosis if CSF is collected too early, or too late. Extrapolating from the human experience, several of our dogs had sub-acute to chronic clinical disease (e.g. cases 13 and 22). In these patients, the initial florid inflammatory response may have dissipated by the time CSF was collected. If signs were indeed referable to late sequelae, the poor response to treatment would be explicable.

Eight of the 10 dogs in the contemporary cohort tested had a peripheral eosinophilia and similar observations were made in Mason's series, although he did not reproduce this in experimentally-infected dogs [14]. All dogs in Mason's series with peripheral eosinophilia also had intestinal parasites. None of the dogs in our contemporary series had faecal samples examined for parasites. In humans, peripheral eosinophilia is commonly seen with NA and these patients generally have no evidence of intestinal parasitism [9]. Not all ingested larvae reach the host's CNS, so some must die in other tissues, presumably inciting an eosinophilic granulomatous reaction [1, 13, 14, 47]. Such a mechanism would account for the peripheral eosinophilia noted in our series.

Advanced imaging is useful in human NA [63–65], but has yet to be of proven benefit in canine NA. Three of the prospective cohort had myelography or a CT study (Cases 1, 18 and 19) but no definitive abnormalities were detected. In human patients, MRI appears to be the superior imaging modality for diagnosing NA, although multi-slice helical CT can sometimes resolve verminous granulomas e.g. along the optic nerve. High field MRI scanners (> 1.5 T) using paramagnetic contrast agents can display images of individual larvae and/or migration tracts in the neural parenchyma [12]. Lesions may not be evident at the time of presentation and actually can be most visible after the patient has begun to improve, taking many months to resolve [65–67].

Some parasitic infections can be diagnosed by demonstrating patency. Specifically for *A. vasorum*, faecal analysis using the Baerman technique yields L_1_ in faeces and bronchial washings of symptomatic dogs and foxes because parasites matures to the reproductive stage [68]. However, as *A. cantonensis* can only become patent in rats [13, 45, 47, 69–71], faecal analysis is of no value for diagnosing canine NA.

### Detection of anti-*A. cantonensis* antibodies

#### Serum ELISA

ELISA testing of serum using a crude antigen preparation was not sensitive or specific at diagnosing NA. Only 12/19 dogs (63%) with NA had positive serum titres, compared to 28/43 (65%) of control dogs. Two dogs with NA (Cases 1 and 3) had serum samples tested one year after initial presentation; both remained positive, albeit with low titres. Thus, IgG titres likely persist for several months following infection (although the dogs might have been re-exposed). It is possible that some control dogs were positive because they had been previously exposed to *A. cantonensis*, developing subclinical self-limiting infections and a successful immune response.

A 1980 seroepidemiologic study suggested a substantial level of subclinical *A. cantonensis* infections in Aboriginal people in northern Australia [72], but lack of controls and use of a crude antigen for testing raised concerns about the validity of these findings. Indeed, a high level of cross-reaction with other nematode infections (e.g. *Stongyloides stercoralis*, visceral larval migrans of *Toxocara canis*) seems more likely [62]. The use of a crude antigen in our ELISA would also have the potential for a significant level of cross-reaction when using serum, the high percentage of control cases with positive titres simply reflecting cross-reaction with other helminths (*Toxocara canis, Ancylostoma caninum, Trichuris vulpis*, etc.), especially in young dogs. In a serological study of NA in macropods using the same ELISA, a substantial number of false positives in control groups were observed from macropods in areas where *A. cantonensis* is not considered endemic [25]. Again, the most likely explanation was cross-reaction with other nematodes. For this reason, numerous immunologic tests for NA have been abandoned, including indirect haemaggultination [73], intradermal testing [74] and complement fixation [75]. At least 25 antigens from adult *A. cantonensis* are shared with other helminths [76] and substantial cross reactivity exists amongst larval stages [28, 77, 78]. The variability of anti-*A. cantonensis* IgG titres in serum of our NA cases probably has, in part, a similar explanation.

#### CSF ELISA and Western blots

ELISA testing using CSF appears much more promising as a means of diagnosing NA. Antibodies against *A. cantonensis* in CSF are expected to be incited by the presence of larvae within the CNS and produced locally, as the blood brain barrier is normally impermeable to IgG in the circulation. Positive results were observed in 19/21 NA cases (90%), but in only one of the control group (a CSF specimen contaminated by blood during collection). The latter chance finding emphasises that high IgG levels in serum may give rise to false positive results if there is contamination of CSF with blood. Testing of CSF samples from dogs with other inflammatory or neoplastic neurological diseases would be useful to further determine the sensitivity and specificity of the ELISA. Four dogs with intervertebral disc disease (confirmed by myelography and surgery) had CSF tested using the ELISA and were negative (data not shown). A larger number of specimens from cases with other CNS diseases would make the observation more compelling.

All cases in the prospective cohort tested using Western Blot analysis (14 cases) were positive. Interestingly, dogs did not make IgG to the 204 kDa antigen but reacted strongly to the 31 kDa antigen, while macropods reacted to both antigens (like rats and human patients) [25, 31]. Unfortunately, no CSF specimens from control dogs were subjected to Western blot analysis, but control macropod serum tested negative, as expected. Using macropod serum, Western blots using the 31 kDa antigen had a sensitivity of 62% and specificity of 92% [25]. The most critical limitation of Western blot testing in the macropod study was the number of false negatives, but the rarity of false positives suggests its use as a confirmatory technique in dogs with suspect NA is valid.

The recent development of a genus specific polymerase chain reaction (PCR) [59] and loop-mediated isothermal amplification (LAMP) [79] tests for *Angiostrongylus* species is of great interest, and such a test might be suitable for diagnosing of NA using whole blood, CSF or tissue obtained at necropsy. PCR testing is already proving useful for diagnosis of *A. vasorum* infections in dogs in the UK [59, 80].

### Treatment

#### Role of glucocorticoids

All dogs in the prospective cohort received glucocorticoids. Reducing inflammation associated with dead or moulting *A. cantonensis* larvae should improve signs in dogs with eosinophilic meningo-encephalomyelitis. This was certainly true for our cases, except in four dogs (described below). The response to glucocorticoids was rapid and most dogs improved substantially within two days. In particular, hyperaesthesia was conspicuously improved. In contrast, the historical cohort demonstrated a very high rate of mortality despite glucocorticoid therapy, possibly because of heavier infections in immunologically näive patients with small vertebral canals and spinal cords.

In dogs that ingest numerous larvae, using corticosteroids to dampen the immune response may not, in itself, result in a favourable outcome. In fact, the disease may progress due to continued larval migration, unhindered by the host's immune response. Several dogs in the historical cohort worsened despite treatment with glucocorticoids, possibly due to this phenomenon. Clearly, mechanical damage due to migrating larvae and inflammation secondary to the appropriate host immune response both contribute to neural damage [43, 66, 81–85]. Which of the two is the more significant depends on (i) the age, species and breed of the infected host, (ii) the number of larvae ingested and the time course over which infection occurs, (iii) the physical dimensions of the spinal cord and nerve roots. In very young dogs, like those in Mason's series, it may be that the immune system is simply overwhelmed and the large number of migrating larvae (and secondary inflammation) produce extensive trauma to neural tissues and such pups die or must be euthanased. Similar considerations apply to an even greater extent in tawny frogmouths (an owl or jay-like Australian native bird) because of their extremely small spinal cord.

Of the four patients in the contemporary cohort that did not respond to glucocorticoids therapy, two dogs (Cases 13 and 22) presented for chronic progressive CNS signs including behavioural changes. Both were eventually euthanased due to intractable aggression that developed after treatment. Another dog (Case 18) had a 6-month history of head tremors unresponsive to therapy, while a fourth dog (Case 19) presented for cranial nerve dysfunction, cervical pain, progressing to blindness. Unfortunately, necropsy confirmation was not possible in these cases, which limits inferences that can be made. Indeed, it is possible that these dogs had other CNS diseases. In the retrospective cohort, a number of pups failed to fully recover and had persistent hind limb paresis. Permanent sequelae are seen in human NA, particularly in children, and include blindness, altered mentation and facial nerve palsies [35, 60]. Jindrak was able to demonstrate, in dogs, that granulomas associated with larval fragments were present in the CNS 60 days post-infection [13], but by 90 days, lesions were no longer evident. In humans, MRI demonstrate lesions within the CNS for up to 22 weeks post-infection [65]. Thus, the length of corticosteroid therapy should continue for at least 6 to 8 weeks, although this may need to be extended.

There are potential complications associated with long-term administration of corticosteroids. Two dogs in the contemporary group (Cases 2 and 3) were smaller than litter mates at the cessation of treatment, and at maturity appeared under-sized for their respective breeds. Studies on the growth of children receiving corticosteroids have shown that some fail to grow to their expected height [86]. Secondary infections are another complication of long-term corticosteroid use. Case 4 developed demodectic mange during treatment, which was successfully managed with topical therapy. Translocated enteric bacterial infections have been described secondary to parasites migrating from the GIT [87]. Long-term glucocorticoid therapy would further increase the risk of infections, thus broad-spectrum antibiotics are justified in these patients.

#### Role of anthelmintics

There is a history of scepticism regarding the place of anthelmintics in managing veterinary NA cases. If pathology is due predominantly to the host's reaction to larvae, then giving anthelmintics soon after infection should hasten the onset of signs. Similarly, administering anthelmintics during the natural course of the disease might worsen the inflammatory response. In Mason's study, 8 of 55 pups received anthelmintics without concomitant glucocorticoids, all amongst the most severely affected of the cohort; six died or were euthanased and the remaining two pups had prolonged recoveries [14]. The conclusion was that anthelmintics worsened clinical outcomes, because they synchronise the release of metazoan antigens.

In human medicine, the practice of administering anthelmintics is an accepted part of therapy for NA in many Asian countries. The rationale is that in severe cases there is a significant component of neural damage due to mechanical injury by migrating larvae [16]. There is also evidence that vascular lesions (thrombosis, aneurysms) occur secondary to larval migration [16]. Anthelmintics have been used successfully in other species to treat NA [88, 89]. Two cases (see Additional file 4: Appendix 4) that did not meet the inclusion criteria for our study are worthy of comment. In a litter of seven Greyhound pups with multifocal CNS disease, six died despite glucocorticoid therapy, but a single pup treated with fenbendazole as well as glucocorticoids recovered. Necropsy of one of the littermates that died demonstrated numerous *A. cantonensis* larvae within the cord, brain and meninges. The second case, a 3-month female Boxer, had been treated with corticosteroids and ivermectin for three weeks for presumptive NA. At referral, the dog had eosinophilic pleocytosis in CSF (111 cells/μL; 85% eosinophils). Ivermectin was stopped. Although the dog initially improved over a 2-week period, seven weeks after referral it relapsed despite a tapering glucocorticoid regimen and was euthanased. Necropsy revealed *A. cantonensis* larvae within the spinal cord and brain. These cases suggest that administration of anthelmintics may be useful in treating NA, if combined with corticosteroids and other immune modulators. Experimentally, immunosuppression reduces meningitic signs but worsens myelo-encephalitic disease by inhibiting eosinophil function [85]. The use of anthelmintics therefore seems cogent [82, 90]. An escalating dosage regimen that kills larvae slowly and non-synchronously, in conjunction with glucocorticoids, has theoretical merit. Such an approach has been used successfully in a recent paediatric patient treated in Sydney in 2011 (Professor Alison Kesson, personal communication).

#### Adjunctive therapy

All dogs in our study received symptomatic and supportive care. Such care is critical, as is management of hyperaesthesia. Mu opioid agonists (e.g. morphine, methadone, buprenorphine) are useful adjuncts to glucocorticoids for treating hyperaesthesia [91] and drugs effective for neuropathic pain (gabapentin, pregabalin) might also be useful adjuncts. Manual expression of the bladder or urinary catheterisation may be required in some instances. Padded bedding is important for severely affected patients, as is nursing in sternal recumbency to optimise lung function. Fluid therapy is indicated if the dog cannot drink or if there is a risk of aspiration. Physiotherapy and massage during convalescence will help minimise muscle wasting or contracture, although care must be taken with hyperaesthetic animals.

Cyclosporine was helpful in a mouse model of NA [92]. The benefits appear to be twofold: i) its effect on T cells reduces the host's immune response, particularly eosinophil recruitment and ii) it has a direct anti-parasitic effect. The micro-emulsion formulation of cyclosporine has reduced the need for drug monitoring and a dose of 5 mg/kg daily is safe in virtually all dogs [93]. To date, there are no reports of human cases receiving cyclosporine. Other drugs that modify the T_H_1 and T_H_2 responses may also be beneficial, such as tumour necrosis factor-α inhibitors, e.g. thalidomide [94]. Matrix metalloproteinases play a critical role in CNS inflammation [83, 95] and their inhibition reduced the severity of NA lesions in mice [96, 97]. A number of drugs, including doxycycline and albendazole, inhibit metalloproteinases and such agents may prove useful in treating NA [90, 96].

#### Prevention

In other species, acquired immunity appears to be short lived or incapable of preventing re-infection [98–101]. Firm recommendations cannot be made with regard to preventing NA in dogs using anthelmintics. There is no evidence that routine heartworm or intestinal prophylaxis prevents NA, although they do prevent patent infections with the closely related *A. vasorum*. Most anthelmintics used for routine heartworm or intestinal prophylaxis are administered monthly and most drugs (ivermectin, selamectin, milbemycin) attain therapeutic levels in blood for less than 48 h. Because *A. cantonensis* larvae can be seen in the CNS within hours to days following ingestion [1], anthelmintics would need to be present in circulation continuously to be an effective prophylactic. Following dermal administration of a combination product (Advocate; Bayer) containing moxidectin and imidacloprid, moxidectin takes 4-10 days for maximum concentrations in serum to be reached, with substantial levels persisting in blood for 28 days. Thus this combination "spot on" product, if given frequently enough (perhaps every 2 weeks), or the moxidectin in depot formulation (Proheart; Pfizer Animal Health), might provide effective prophylaxis. Experimental studies looking at the efficacy of such products in this setting would be helpful.

Environmental control of definitive and intermediate hosts represents the most effective approach to reducing the likelihood of infection. Preventing contamination of food and food containers, clearing out areas that might harbour rats, limiting access to gardens where slugs and snails may reside and good hygiene would all limit exposure of dogs to potential sources of infection. This would also reduce the risk to clients and their children. Further, the safe use of anti-coagulant rodenticides (to reduce rat numbers) and molluscicides (to reduce snail and slug numbers) in the environment should minimise the risk to adult dogs. For puppies, perhaps the best prevention is to keep them indoors or under close supervision outdoors. It might be prudent to dose pups with moxidectin should they be presented within 1-2 days of eating slugs or snails. Importantly, dogs with NA should be considered sentinels in the prevention of human NA.

#### Comparisons with A. vasorum

In the UK, *A. vasorum* has behaved as an emerging infectious disease over the last 15 years or so, becoming more prevalent overall and extending its natural range into new districts from focal enzootic foci [59, 80, 102, 103]. Historically, the disease was most common in the south, but its range is extending northwards into Scotland [102]. It is generally thought that both foxes and snails play a critical part in the epidemiology of this disease. Perhaps global warming or changes in the epidemiology of the UK canid population, including its genetic constitution and movement of pets back and forth from Europe may have changed the distribution of cases and clinical spectrum of disease observed in the UK [102].

In Australia, the geographic range of *A. cantonensis* is extending southwards. In the 1970s, it was considered a tropical disease seen only in Queensland, whereas now it is seen commonly in New South Wales. Surveys of rats and molluscs have established that the range of *A. cantonensis* extends at least as far south as Jervis Bay on the south coast of New South Wales [104], although it seems likely that the distribution of this parasite will spread further south. In Australia, pet dogs and tawny frogmouths appear to be the two key sentinel species. In Sydney, disease was first reported in dogs in 1992, whereas the first human cases were reported in 2003 [105]. We have recently heard anecdotal data suggesting NA is present in dogs in Samoa (Belinda Hamlin, personal communication), and it seems likely it is present but currently unrecognised in other Pacific Islands (Micronesia, Polynesia, Hawaii), south east Asia, the Caribbean and possibly even Louisiana [58, 106, 107].

There are many parallels between the epidemiology of *A. vasorum* and *A. cantonensis* in the UK and Australia, respectively, and likely we can learn more (e.g. about molecular diagnostics and strategies concerning disease prevention) by continuing to compare and contrast these two clinical entities.

## Endnotes

^A^ Four additional cases in Collins *et al.*[32] from Sydney had adequate clinical and demographic data, but CSF was not collected. Two 12-week-old German shepherd pups were presented for generalised hyperaesthesia and hind limb weakness. They developed pyrexia and diarrhoea and were treated with flunixin and an anti-diarrheal mixture and made a complete recovery. Three weeks later, two more puppies, aged 5-weeks, presented for generalised hyperaesthesia, diarrhoea and hind limb weakness and died soon thereafter. *A. cantonensis* larvae were recovered from the spinal cords and cerebellum of both puppies in association with eosinophilic granulomatous inflammation.

**Figure 6:**
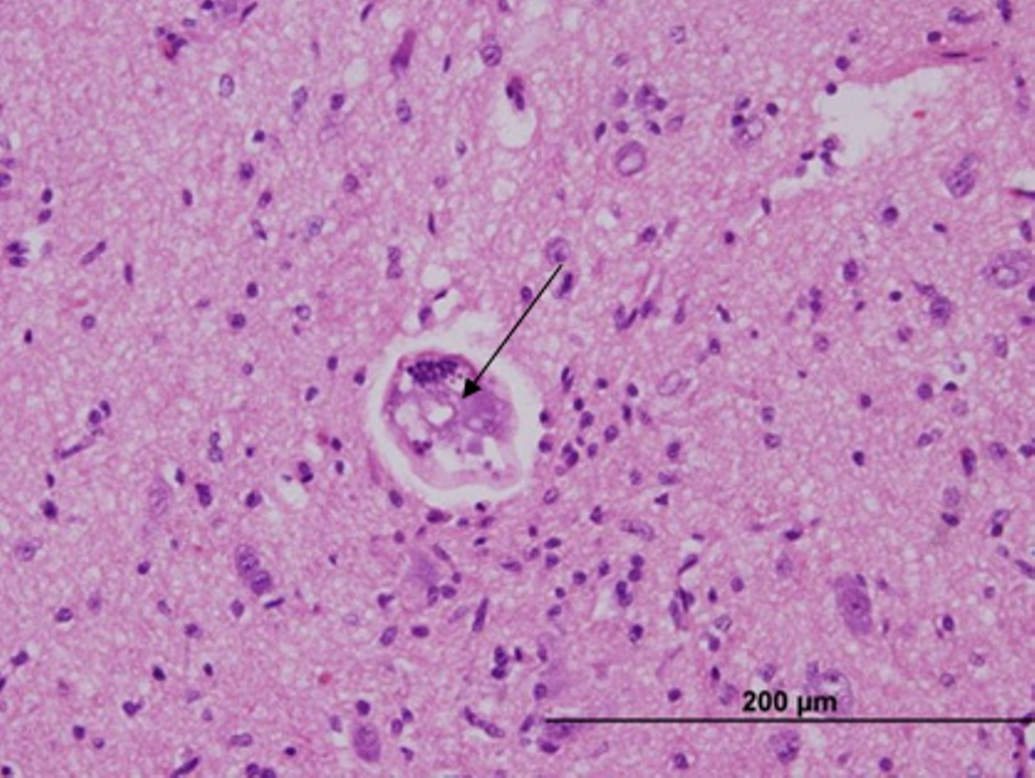
**Haematoxylin and eosin stained section of the brain of a greyhound pup that died of neural angiostrongylosis**. Note the nematode larvae (arrow) in transverse section on the middle of the neuropile.

^B^ A litter of six greyhound puppies with NA did not meet the entry criteria as CSF specimens were unavailable. Five developed hind limb paralysis, urinary and faecal incontinence and severe hyperaesthesia. Four died within three days of developing signs. At necropsy, one pup had granulomatous encephalitis with the occasional nematode within the granulomas (Figure 6). The dogs had been moved to a new kennel, which was near a former rats nest, and there were many snails around the dogs' run.

## Electronic supplementary material

Additional file 1: Appendix 1. Letter sent to all registered Small Animal Specialists and Veterinary Pathologists in Queensland and New South Wales. (DOC 48 KB)

Additional file 2: Appendix 2. Questionnaire sent to clinicians with suspect cases of NA. (DOC 66 KB)

Additional file 3: **Appendix 3**. ELISA Methods: Antigen Production {TC "ELISA Methods: Antigen Production" \f C \l "3"} [108, 109]. (DOC 52 KB)

Additional file 4: Appendix 4. Control Group B Case Details. (DOC 62 KB)

Additional file 5: **Appendix 5**. Case Details: Outline below are the case details from Mason Master's thesis published in 1983 [14]. (DOC 866 KB)

Additional file 6: Appendix 6. (A) Comparison between published and contemporary cases as they occurred by month. (B) Comparison between Sydney and Brisbane cases (pooled recorded and contemporary) as they occurred by month. Mean daily temperature, humidity and rainfall for the Sydney (C) and Brisbane (D) regions. Source: Australian Bureau of Meteorology, 2005. (DOC 62 KB)

Additional file 7: Appendix 7. Case Details. (DOC 68 KB)

Below are the links to the authors’ original submitted files for images.

Authors’ original file for figure 1

Authors’ original file for figure 2

Authors’ original file for figure 3

Authors’ original file for figure 4

Authors’ original file for figure 5

Authors’ original file for figure 6

Authors’ original file for figure 7
