# Supplementary material for: Twenty two cases of canine neural angiostrongylosis in eastern Australia (2002-2005) and a review of the literature
Source: Parasit Vectors. 2012 Apr 5;5:70. doi: 10.1186/1756-3305-5-70 (PMC3361490; doi:10.1186/1756-3305-5-70)
Supplement: Supplementary file 1 — Additional file 1: Appendix 1. Letter sent to all registered Small Animal Specialists and Veterinary Pathologists in Queensland and New South Wales. (DOC 48 KB) [file 13071_2011_563_MOESM1_ESM.DOC]

### Supplementary Material

### Appendix 1

Letter sent to all registered Small Animal Specialists and Veterinary Pathologists in Queensland and New South Wales

«Title» «FirstName» «LastName»

«Company»

«Address1»

Monday 19^th^ May 2003

#### Re: Eosinophilic Meningoencephalitis: neural angiostrongylosis

Dear «Title» «LastName»,

I am currently undertaking a Masters in Veterinary Clinical Studies at the Veterinary Teaching Hospital, University of Sydney. The main aim of the project is to improve an ELISA used to diagnose canine neural angiostrongylosis (NA). Characteristically, the disease is associated with:

- Young dogs
- History of contact with infected slugs and snails.
- Progressive clinical signs characterised by ascending CNS (UMN or LMN) dysfunction and hyperaesthesia.
- Peripheral eosinophilia
- Marked eosinophilic pleocytosis of CSF.
- Improvement after treatment with corticosteroids.

I would greatly appreciate your help in identifying any suspected cases of NA that you may have or have seen in your practice.

Ideally I would require the history with particular regard to onset of clinical signs post contact with slugs/snails (infected material) and their worming/heartworm prophylaxis status as well as any clinical pathology reports. Serum and CSF samples would be particularly helpful. If possible serial serum samples two weeks apart would be very useful.

Samples from cases that do not appear to be due to infection with *A. cantonensis* but have similar signalment or presentation would be useful in testing the validity of the ELISA or identifying atypical cases.

Currently, the ELISA takes 2 weeks to run and would not greatly facilitate case management, but we are hoping to ultimately reduce the time interval. If you have any questions or require any more information please contact me at the Veterinary Teaching Hospital either by phone: (02) 9351 3437; or email: [jalunn@vetc.usyd.edu.au](mailto:jalunn@vetc.usyd.edu.au). Please do not hesitate to contact me if you have any further questions.

Yours sincerely,

Julian Lunn BVSc MACVSc

University Veterinary Centre, Sydney

Faculty of Veterinary Science

University of Sydney

NSW 2006
