# Supplementary material for: Twenty two cases of canine neural angiostrongylosis in eastern Australia (2002-2005) and a review of the literature
Source: Parasit Vectors. 2012 Apr 5;5:70. doi: 10.1186/1756-3305-5-70 (PMC3361490; doi:10.1186/1756-3305-5-70)
Supplement: Supplementary file 3 — Additional file 3: Appendix 3. ELISA Methods: Antigen Production {TC "ELISA Methods: Antigen Production" \f C \l "3"} [108, 109]. (DOC 52 KB) [file 13071_2011_563_MOESM3_ESM.DOC]

**Appendix 3**

# ELISA Methods: Antigen Production

***Obtaining* A. cantonensis *larvae***

*A. cantonensis* larval antigen was obtained from the Department of Parasitology, Institute of Clinical Pathology and Medical Research (ICPMR) at Westmead Hospital. The life cycle of *A. cantonensis* was sustained using fresh water snails (*Biomphalaria glabrata*) which were infected through ingestion of plant material contaminated with rat faeces. The rat faeces were collected from Wister rats (*Rattus norvegicus*), housed in the Department of Parasitology, that had already been infected with *A. cantonensis* and contained 1^st^ stage larvae confirmed by the Baerman technique.

Snails were examined at 4 weeks post-exposure to confirm the presence of infective 3^rd^ stage larvae. Wistar rats were then infected by ingestion of the infected snails. This process was facilitated by sedation of a rat with carbon dioxide and introduction of a fresh snail carcass into the rat’s mouth. Upon recovery from the sedation the rat ingested the snail. Faeces were collected 2 weeks after infection and examined for the presence of 1^st^ stage larvae. The infected rats showed only mild respiratory signs when initially infected and could be maintained for a number of months readily producing large numbers of 1^st^ stage larvae in the faeces. Weight loss was the most significant clinical finding in the long term, although some rats died from respiratory disease.

A single, infected female Wistar rat was euthanased by inhaling carbon dioxide. At necropsy 13 female and 9 male *A. cantonensis* adults were recovered from the right ventricle, pulmonary arteries and bronchi for antigen preparation

## Preparation of antigen

Antigen from *A. cantonensis* adults was prepared by the Department of Parasitology, ICPMR. Adults recovered as described above were washed in phosphate buffered saline (PBS) (Appendix 3) at room temperature. The process was repeated 3 times. Whole adults were frozen to –70°C in a refrigerator then subsequently freeze-dried to –196°C using liquid nitrogen. The freeze-dried adults were then homogenised with a mortar and pestle in 10 mL of anti-protease buffer (Appendix 3). The resulting solution was stored at 4°C for 24 hours to facilitate the extraction of the proteins. The solution was the centrifuged at 3000g for 15 minutes and the resultant supernatant extracted. The supernatant was centrifuged at 100,000g for 90 minutes at 4°C (Beckman Refrigerated Ultracentrifuge). The resulting supernatant containing the adult antigens was decanted and stored in 500 µL aliquots at –70°C.

The protein concentration was determined using established techniques.[102] A Pierce Bicinchoninic Acid Protein Assay kit was used (Pierce®, Rockford, IL, USA).

# Secondary Antibodies

A commercially available anti-dog IgG was used as the secondary antibody. Antibodies to canine Immunoglobulin was generated in rabbits using affinity purified canine IgG (heavy and light chain specific) in a two-step glutaraldehyde method.[103] The rabbit anti-dog IgG was conjugated to horseradish peroxidase (HRP, Sigma®, St Louise, Missouri, USA).

# Substrate

Tetramethyl benzidine (TMB) (K-BLUE substrate, ELISA Systems) was used as the substrate for the HRP in the ELISA. One molar phosphoric acid (AnalaR®, BHD Laboratory Supplies, Poole, England) was used to stop the enzymic reaction.

# Phosphate Buffered Saline

Sodium Chloride 160 mg

Potassium Chloride 4 g

Sodium Hydrogen Orthophosphate 23 g

Potassium Dihydrogen Orthophosphate 4 g

Salts were added to two litres of distilled water until completely dissolved. A dilution of 1 in 100 was used as required.

**Anti-protease Buffer**

Aprotinin (Trasylol, Bayer®)

Phenylmethylsulfonyl fluoride (PMSF) (Sigma®)

**Blocking Buffer**

Milk powder (Diploma, Australia) 5 g

Phosphate buffered saline (PBS) 100 mL

Tween 20 50 µL

**Blotto**

Milk powder (Diploma, Australia) 2 g

Tween 20 100 µL

Sterile water 100 mL

**Chromagen Solution**

4-chloronapthol 0.008 g

Methanol 4 mL

H_2_O_2_ 10 µL

PBS 10 mL
