# Supplementary material for: Twenty two cases of canine neural angiostrongylosis in eastern Australia (2002-2005) and a review of the literature
Source: Parasit Vectors. 2012 Apr 5;5:70. doi: 10.1186/1756-3305-5-70 (PMC3361490; doi:10.1186/1756-3305-5-70)
Supplement: Supplementary file 4 — Additional file 4: Appendix 4. Control Group B Case Details. (DOC 62 KB) [file 13071_2011_563_MOESM4_ESM.DOC]

### Appendix 4

**Control Group B Case Details.** All samples were collected from dogs with no appreciable CNS dysfunction undergoing desexing or orthopaedic procedures during May 2005.

| **Control Group B – Serum Controls** | | | |
| --- | --- | --- | --- |
| **Sample No.** | **Gender** | **Age (weeks)** | **Breed** |
| 1 | MN | 208 | Doberman cross |
| 2 | FN | 416 | Curly coated Retriever |
| 3 | MN | 468 | Dalmatian |
| 4 | MN | 312 | Malamute |
| 5 | ME | 48 | Staffordshire Bull Terrier |
| 6 | ME | 36 | Papillion |
| 7 | FE | 260 | Staffordshire Bull Terrier |
| 8 | FN | 208 | German Shepherd |
| 9 | ME | 24 | Labrador |
| 10 | ME | 20 | Yorkshire Terrier |
| 11 | FE | 28 | Hungarian Vizla |
| 12 | MN | 468 | Kelpie |
| 13 | MN | 624 | Mastiff cross |
| 14 | MN | 48 | Boxer |
| 15 | MN | 40 | Golden Retriever |
| 16 | MN | 156 | German Shepherd |
| 17 | MN | 156 | Staffordshire Bull Terrier |
| 18 | FN | 468 | German Shepherd |
| 19 | FE | 104 | Doberman |
| 20 | FN | 624 | Cocker Spaniel |
| 21 | MN | 312 | Labrador cross |
| 22 | MN | 324 | Labrador |
|  | **Median age** | **208** |  |
|  |  |  |  |

Control Group B.

MN = castrated male, ME = entire male, FN = spayed female, FE = entire female.
