# Supplementary material for: Twenty two cases of canine neural angiostrongylosis in eastern Australia (2002-2005) and a review of the literature
Source: Parasit Vectors. 2012 Apr 5;5:70. doi: 10.1186/1756-3305-5-70 (PMC3361490; doi:10.1186/1756-3305-5-70)
Supplement: Supplementary file 5 — Additional file 5: Appendix 5. Case Details: Outline below are the case details from Mason Master's thesis published in 1983 [14]. (DOC 866 KB) [file 13071_2011_563_MOESM5_ESM.DOC]

### Appendix 5

# Case Details

Outlined below are the case details from Mason Master’s thesis published in 1983.[14]

### *
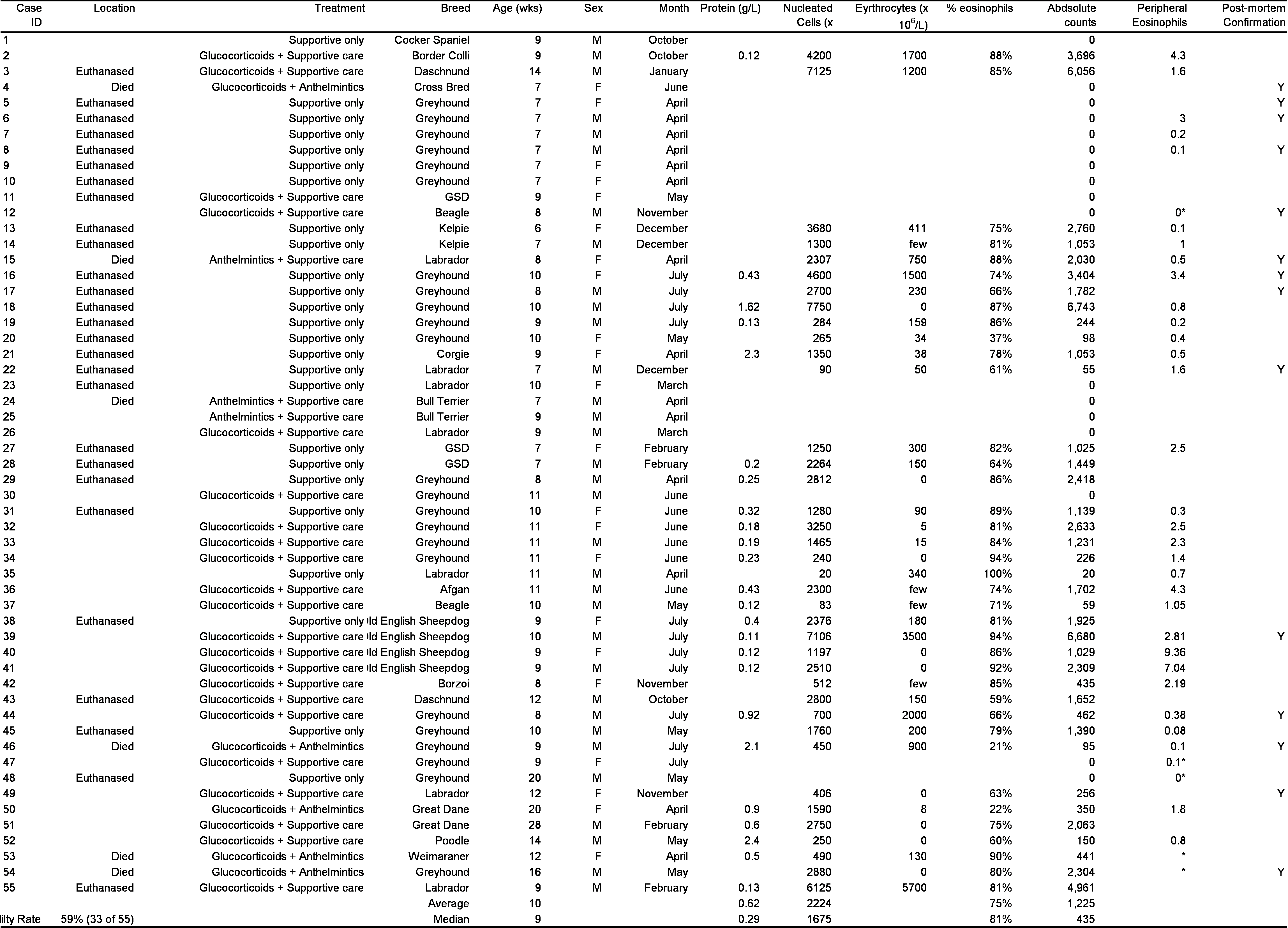
*
