# Supplementary material for: Twenty two cases of canine neural angiostrongylosis in eastern Australia (2002-2005) and a review of the literature
Source: Parasit Vectors. 2012 Apr 5;5:70. doi: 10.1186/1756-3305-5-70 (PMC3361490; doi:10.1186/1756-3305-5-70)
Supplement: Supplementary file 6 — Additional file 6: Appendix 6. (A) Comparison between published and contemporary cases as they occurred by month. (B) Comparison between Sydney and Brisbane cases (pooled recorded and contemporary) as they occurred by month. Mean daily temperature, humidity and rainfall for the Sydney (C) and Brisbane (D) regions. Source: Australian Bureau of Meteorology, 2005. (DOC 62 KB) [file 13071_2011_563_MOESM6_ESM.DOC]

**A**

#

**B**

**C**

**D**

**Appendix 7. (A) Comparison between published and contemporary cases as they occurred by month. (B) Comparison between Sydney and Brisbane cases (pooled recorded and contemporary) as they occurred by month. Mean daily temperature, humidity and rainfall for the Sydney (C) and Brisbane (D) regions. Source: Australian Bureau of Meteorology, 2005.**
