# Supplementary material for: Twenty two cases of canine neural angiostrongylosis in eastern Australia (2002-2005) and a review of the literature
Source: Parasit Vectors. 2012 Apr 5;5:70. doi: 10.1186/1756-3305-5-70 (PMC3361490; doi:10.1186/1756-3305-5-70)
Supplement: Supplementary file 7 — Additional file 7: Appendix 7. Case Details. (DOC 68 KB) [file 13071_2011_563_MOESM7_ESM.DOC]

### Appendix 6

# Case Details

***Case 1: Labrador retriever, 6-month-old MN (Eastwood, NSW, May, 2004)***

The dog was presented for lethargy and mild neck pain. There was no history of slug or snail ingestion. Signs progressed to severe neck pain and forelimb hyperaesthesia. There was no improvement with NSAID therapy. The most prominent haematologic abnormality was marked eosinophilia. CSF analysis revealed eosinophilic pleocytosis. Glucocorticoid therapy was started and the dog improved within 24 hours. The dog had been given an injectable heartworm prophylaxis given 2 months before onset of clinical signs. Therapy was continued for 8 weeks. The dog made a full recovery.

***Case 2: Golden Retriever, 5-month-old FE (Bellevue Hill, NSW, May, 2002)***

The bitch was presented for hind limb ataxia and urinary/faecal incontinence. Dog had been known to eat slugs and snails. Two days prior to presentation, the owners noticed that the dog had become ataxic in the hind limbs and was urinating and defecating in the house, which it had not done before. On presentation, the dog could stand but was paraparetic with proprioceptive deficits, reduced patella reflexes and mild hind limb muscle atrophy, worse on the left. The bladder was easily expressed and the dog was hyperaesthetic around the lumbar region. The dog had been given topical imidacloprid and ivermectin 5 days before the onset of clinical signs. Following CSF collection, the dog was treated with intravenous glucocorticoids initially. The dog improved significantly over 24 hours and was able to walk unassisted. Seven days after presentation the incontinence was still evident, although in other respects the dog was substantially better with no lumbar hyperaesthesia and only mild hind limb proprioceptive deficits. The dose of glucocorticoids was reduced from 1mg/kg BID to 0.5 mg/kg, but signs worsened over the subsequent 7 days and the dose was increased to the original level. Three weeks after initial presentation the dog began to vomit, which was treated with dietary modification. The neurological exam was normal and the dose of glucocorticoids was tapered. Two months following the initial diagnosis the dog developed focal demodecosis and the prednisolone was stopped. The dog was subsequently treated successfully with topical amitraz.

***Case 3: Kelpie, 6-month-old FN (Redfern, NSW, May, 2001)***

The bitch was presented 5 days after routine spay for lethargy. The most significant physical finding was pain on manipulation of the tail and during a rectal exam. The dog had been seen eating slugs and snails on a number of occasions. Hyperaesthesia progressed to involve the hind legs and lumbar region over the next 3 days. Initially, there were no proprioceptive deficits in either hind leg. The following day the dog had neck pain and was ataxic in the hind legs. Haematology showed mild eosinophilia. CSF revealed eosinophilic pleocytosis. The dog was treated with glucocorticoids and improved greatly over the following 24 hours. The dog was clinically normal 6 weeks after the initial presentation. Glucocorticoids were stopped after 3 months.

***Case 4: Golden Retriever, 4-month-old FE (Oyster Bay, NSW, March, 2002)***

The bitch was presented for sudden onset of hind limb paresis and urinary incontinence. Signs had progressively worsened over 3 days prior to presentation. The dog was unable to stand on its hind legs and was painful around the hindquarters and lumbar regions. The bladder was easily expressible. There was obvious hind limb muscle atrophy bilaterally, reduced to absent proprioception and increased patella tendon reflexes, the left more exaggerated than the right. Haematology and serum biochemistry were unremarkable. Within 12 hours, its neurologic status had deteriorated: forelimb weakness was now evident, anal tone and hind limb proprioception were absent and there was clonus associated with both patella reflexes. The dog had reduced pupillary light reflexes and shied away from light. CSF analysis revealed an eosinophilic pleocytosis. Glucocorticoid therapy was commenced, and within 12 hours the dog had improved substantially and was now able to stand and appeared much less painful. Over the next 7 days the patient continued to improve although the urinary incontinence and posterior ataxia persisted for a fortnight. Glucocorticoids were discontinued after 4 weeks.

***Case 5: Boxer, 4-month-old FE (Balgowlah, NSW, May, 2002)***

The bitch was presented for a 3-day history of lumbar spinal pain and intermittent diarrhoea. The most striking physical finding was pain upon cervical ventroflexion. Biochemistry and haematology were unremarkable. Neurologically, the dog appeared normal apart from cervical pain. CSF analysis revealed an eosinophilic pleocytosis. There was no serological evidence for a *Neospora caninum* infection, and no abnormalities in spinal radiographs. Glucocorticoid therapy was commenced in concert with clindamycin. The dog was clinically normal within 2 days. Glucocorticoid therapy continued for a further 5 weeks.

***Case 6: Miniature Poodle, 4-month-old ME (St Ives, NSW, May, 2002***

The dog was presented 10 days after routine vaccination with hind limb weakness and severe generalised hyperaesthesia, affecting especially the cervical and lumbar spine. Signs had begun 3- days before presentation but worsened despite carprofen administration. Physical findings included generalised hyperaesthesia, proprioceptive deficits in both hind legs along, reduced hind limb reflexes and weakness, worse on the left. CSF analysis demonstrated eosinophilic pleocytosis. The dog improved substantially within 12 hours of starting glucocorticoid therapy. Within two days, only lumbar hyperaesthesia was evident. Within one month the dog was outwardly normal and glucocorticoids were continued for six weeks in total.

# *Case 7: Rottweiler X, 4-month-old ME (Albany Creek, QLD, June, 2003)*

# The dog was presented for sudden onset of posterior ataxia and hyperaesthesia. This progressed over 2 days to faecal and urinary incontinence, LMN signs in the hind legs and neck pain. There was no history of ingestion of slugs or snails. Biochemistry and haematology were unremarkable. CSF analysis showed eosinophilic pleocytosis. Glucocorticoid therapy was commenced and the dog made a complete recovery over a 4-week period.

***Case 8: Beagle, 5-month-old FE (Ashgrove, QLD, July 2003)***

This bitch was presented for episodes of “extending the neck” and “crying out in pain” for the previous three days. The dog had been given NSAIDs, but with little improvement. The only pertinent finding on physical exam was neck pain. CSF analysis revealed an eosinophilic pleocytosis. Glucocorticoid therapy was commenced and the dog appeared to be pain free within 24 hours. The patient was treated for three months. There was no reoccurrence of the episodes.

***Case 9: Australian Cattle Dog, 12-month-old FE (Cleveland, QLD, February 2004)***

The bitch was presented for acute onset of hind limb weakness with fever, vomiting and lethargy. Weakness and ataxia progressed over the following 24 hours to involve the fore limbs. Physical findings included lack of menace response, with dilated pupils and abnormal pupillary light reflexes as well as tetraparesis. Blood tests were unremarkable. CSF analysis revealed an eosinophilic pleocytosis. The dog responded to glucocorticoid therapy, becoming ambulatory within 24 hours. Peripheral blindness resolved over 48 hours. Therapy was continued for 3 months.

***Case 10: Staffordshire terrier, 2-month-old FE (Chatswood, NSW, February, 2005)***

The bitch was presented for acute onset of posterior ataxia and lumbar hyperaesthesia. Its owners had noticed that the dog had diarrhoea about 2 weeks before presentation. The owner noted a significant rat problem in the area. One of 7 puppies in the litter, one had died at birth while the others were normal. Physical findings included lethargy, hind limb paresis with hyperaesthesia and an easily expressible urinary bladder associated with incontinence. Blood tests revealed only a mild elevation in CK. The dog progressed over the next 24 hours to the point where its tail was flaccid and it was unable to stand. CSF analysis revealed marked eosinophilic pleocytosis. Treatment commenced with glucocorticoids (1mg/kg prednisolone BID) and clindamycin (75mg PO BID). The dog improved over 24 hours and was less painful. *Toxoplasma gondii* and *Neospora caninum* antibody were negative. The dog made a full recovery although the urinary incontinence persisted for 10 days.

***Case 11: Labrador, 8-month-old FE (Lindfield, NSW, April 2005)***

The dog was presented for decreased activity. The most significant physical finding was cervical pain. The dog had been in a Sydney suburban back yard on the lower North Shore but had also visited the South Coast of NSW. Cervical hyperaesthesia was unresponsive to carprofen. The dog initially demonstrated mild peripheral eosinophilia (1.9 x 10^9^/L; normal less than 1.5 x 10^9^/L). Hyperaesthesia progressed to involve the right forelimb. Rectal temperature remained normal through the clinical course. CSF analysis demonstrated marked eosinophilic pleocytosis. Glucocorticoids were dispensed (0.5mg/kg BID) in concert with doxycycline (5mg/kg PO BID). The dog was significantly better within 24 hours and was clinically normal two weeks after treatment commenced. Glucocorticoids were continued for another six weeks (i.e. eight weeks in total).

***Case 12: Labrador retriever, 4-year-old FN (Avalon, NSW, August, 2003)***

The dog was presented for lethargy and neck pain. There was no history of exposure to slugs or snails. Apart from cervical pain there was occasional asymmetrical facial twitching, which subsequently resolved. Haematology was unremarkable except for mild leukocytosis. Eosinophil numbers were at the high end of the reference interval. Biochemistry was unremarkable apart from a two-fold elevation in CK (339; RI 0–180 IU). The dog responded to opioid analgesia. CSF analysis revealed eosinophilic pleocytosis. Glucocorticoid therapy was commenced and the dog showed some improvement over the next day. The dog subsequently made a full recovery.

***Case 13: Boxer, 12-month-old ME (Banksia, NSW, May, 2002)***

The dog presented with a 2-month history of lethargy, hind limb weakness and altered mentation. It had not been receiving intestinal parasite or heartworm prophylaxis. Physical findings included ataxia affecting all four limbs, with crossed extensor reflexes present in the fore and hind limbs. The dog was weak in all four legs with reduced reflexes. The dog also had reduced menace responses in both eyes. Haematology revealed a mild leukocytosis, with eosinophilia (2.5 x 10^9^ cell/L (RI 0.14 – 1.2). CSF demonstrated an eosinophilic pleocytosis. Serum was collected for cryptococcal and neosporosis serologies and therapy with glucocorticoids and trimethoprim/sulpha were initiated. The dog showed no significant improvement over the subsequent four weeks. Azithromycin was added to the regimen, but was without effect. Neither *Neospora* antibodies nor *Cryptococcus* antigen were detected in serum*.* Two months after the initial visit (4 months after onset of signs) there was some minor improvement in ataxia and proprioception, however the owners had observed aggressive tendencies developing. Faecal examination at this time revealed significant number of *Toxocara canis* eggs. The aggressive behavioural changes worsened over the next 4 months and the dog was euthanased at the owner’s request. CSF collected immediately following euthanasia was normal cytologically. Necropsy was not performed.

# *Case 14: Kelpie, 2-year-old FN (Sunnybank Hills, QLD, July, 2003)*

# The bitch was presented for generalised hyperaesthesia and altered mentation. The owner had noted over a five day period that the dog would suddenly cry in pain, or occasionally run into objects within the house and was inappetant. Physical examination revealed ataxia affecting all four limbs, no menace response and generalised hyperaesthesia. CSF examination showed eosinophilic pleocytosis. Glucocorticoid therapy was commenced that the dog made a rapid recovery.

# *Case 15: Staffordshire terrier, 10-year-old FN (Brisbane, QLD, November, 2002)*

The dog presented initially for a splenic haemangiosarcoma, which was removed and adjunct chemotherapy administered. One month after the chemotherapy had finished, the dog presented with acute onset of severe lumbar hyperaesthesia and paraplegia. Radiographs did not demonstrate vertebral metastatic lesions. CSF analysis revealed an eosinophilic pleocytosis. On further questioning it was revealed that the owners lived near industrial estates and had noticed a large population of rats that infested the nearby areas and their own residence. The dog responded to glucocorticoids and recovered uneventfully.

# *Case 16: Rottweiler X, 4-year-old FN (Erskineville, NSW, April, 2002)*

# The bitch was presented for an acute episode of neck pain unresponsive to carprofen. The only significant physical finding was cervical pain associated with lateral and ventral flexion of the neck, apparently localised to the 1^st^ or 2^nd^ cervical vertebrae. The episodes of pain increased in frequency over the following 3 days and the dog became depressed. Plain radiographs and myelography were unremarkable apart from some mild degenerative changes associated with the caudal cervical vertebrae. CSF analysis revealed a moderate non-specific inflammatory response with only 5% eosinophils. Glucocorticoid therapy was commenced and the dog made a full recovery over two weeks. There was no history of ingestions of slugs or snails.

***Case 17: Maltese, 2-year-old MN (Shailer Park, QLD, March 2004)***

The dog was presented for hyperaesthesia manifest as yelping in pain when being picked up. Physical findings included cervical pain and conscious proprioception deficits in the fore limbs, with mild pyrexia. CSF demonstrated eosinophilic pleocytosis. Glucocorticoid therapy was commenced and the following day the dog appeared normal. Treatment was continued for 8 weeks.

***Case 18: Doberman, 11-month-old FN (Charleville, QLD, June 2004)***

The bitch was presented for episodes of “head tremors”. The first episode was noticed six months previously and they continued to occur over the intervening months, increasing in frequency and duration. At the time of presentation, the patient was experiencing several episodes each day. The physical exam was unremarkable. There was a mild eosinophilia on a peripheral blood smear. CT scan of the head and neck revealed no significant abnormalities. CSF analysis demonstrated a mild eosinophilic pleocytosis. The CSF was negative for *N caninum* and *T gondii* antibodies and cryptococcal antigen. Glucocorticoid therapy was commenced; however the dog did not improve. After 3 weeks azathioprine was added, but there was no improvement. CSF analysis two months after treatment had been commenced was unremarkable despite the dog’s clinical signs.

***Case 19: German Shorthair Pointer, 6-year-old ME (Wynnum, QLD, March 2002)***

The dog was presented with a two week history of bilateral ptosis and anisocoria, unresponsive to topical glucocorticoids and systemic carprofen. For the week prior to presentation, the dog became increasingly depressed and appeared to have altered mentation. The most significant findings on physical examination were neck pain, anisocoria and exaggerated menace responses. The ocular exam was otherwise normal. CSF analysis demonstrated an eosinophilic pleocytosis. Glucocorticoid therapy was commenced and the dog initially responded well. However 7 days following the first visit the dog represented for depression, neck pain, anisocoria and weakness. It appeared to have normal proprioception in all legs despite the ataxia, but the neck pain was more pronounced. The anisocoria was accompanied by ptosis. Intravenous glucocorticoids were given and the dog’s hyperaesthesia improved. Ocular signs progressed, however, and the dog became blind in both eyes over the subsequent 24 hours. The dog was euthanased at the owner’s request. No post mortem was performed.

***Case 20: Rhodesian ridgeback, 8-month-old FE (Rushcutters Bay, NSW, March, 2004)***

The bitch was presented with progressive posterior ataxia, lumbar and cervical hyperaesthesia, and reduced proprioception in the hind legs. Cervical pain was especially prominent. The dog was nervous and shied away from being touched. It had received routine intestinal and heartworm prophylaxis within two weeks of clinical signs developing. The dog initially presented for lethargy and severe neck pain after ingesting rotten turkey eggs on the owner’s property located on the mid-north coast of NSW. On presentation the significant physical findings were neck pain and lumbar pain. Over the following two days the hyperaesthesia worsened despite opioid analgesia and the administration of glucocorticoids. Mild posterior ataxia was observed. The dog was sent home on strict rest and observation. A myelogram was performed one week later following little improvement in the dog’s condition. The most significant myelographic finding was attenuated contrast columns from the level of the 3^rd^ cervical vertebra to cauda equina which was presumed to reflect diffuse swelling of the spinal cord. Following the myelogram the dog was significantly more ataxic with an exaggerated gait and wide based stance. CSF cytology demonstrated an eosinophilic pleocytosis. Glucocorticoids were re-administered. The dog improved over the subsequent weeks although did develop diarrhoea, which resolved with dietary modification.

***Case 21: Rottweiler X, 10-week-old ME (Bundaberg, QLD, May, 2004)***

The dog was presented with acute onset of hind limb weakness and ataxia associated with back pain. The owners had not noticed the dog to eat slugs or snails. Physical findings included lumbar hyperaesthesia, posterior weakness, hind limb proprioceptive deficits, increased patella reflexes and urinary/faecal incontinence. Signs progressed over the following two days to the point where the dog could no longer supports its own weight. Glucocorticoid therapy was commenced and the dog began improving after 24 hours. With supportive therapy the dog made a full recovery.

***Case 22: Bulldog, 4-month-old ME (Cobbitty, NSW, November, 2002)***

The dog had a history of chronic neck pain, behavioural changes, tetraparesis and apparent central blindness that had progressed over two months with little response to carprofen. Physical findings included reduced pupillary light reflexes and diminished sensation from the face and corneas bilaterally. The dog appeared aware of its surroundings but was unresponsive and tended to adopt a wide base stance. Conscious proprioception appeared to be reduced in all four limbs. Limited biochemistry was unremarkable and the dog’s packed red cells and plasma protein concentration were within normal limits. CSF analysis revealed an eosinophilic pleocytosis and the dog was treated with glucocorticoids. Subsequent *Neospora canis* serology was equivocal on serum but negative on CSF. The dog’s ataxia improved over the following 4 weeks however behavioural changes worsened and the dog became more aggressive. The owners declined further workup and the dog was euthanased.

***Positive necropsy with no CSF*: *Greyhound, 8-week-old FE (March, 2003)***

In a litter of 6 puppies, 5 developed hind limb paralysis, urinary and faecal incontinence and severe hyperaesthesia. Four died within three days of the onset of clinical signs and at necropsy, one of puppies had changes suggestive of granulomatous encephalitis with the occasional nematode section seen within the granulomas. The dogs had been moved to a new kennel, which was near a former rats nest, and there were many snails around the dog’s run. The 5^th^ surviving puppy deteriorated despite the use of glucocorticoids. Ivermectin was administered subcutaneously (500 μg/kg) in conjunction with the prednisolone and the puppy improved. There was complete resolution of clinical signs within 2 weeks and the puppy made a full recovery. Glucocorticoids were discontinued after 5 weeks. Histopathology on one of the pups that died demonstrated multiple, large, circumscribed pyogranulomatous foci distributed randomly through the white matter of the lumbar, thoracic and cervical spinal cord and medulla. Nematode sections were seen within some of these pyogranulomas.
