# Supplementary figures and images for: Twenty two cases of canine neural angiostrongylosis in eastern Australia (2002-2005) and a review of the literature
Source: Parasit Vectors. 2012 Apr 5;5:70. doi: 10.1186/1756-3305-5-70 (PMC3361490; doi:10.1186/1756-3305-5-70)

**Optical density (450nm)**

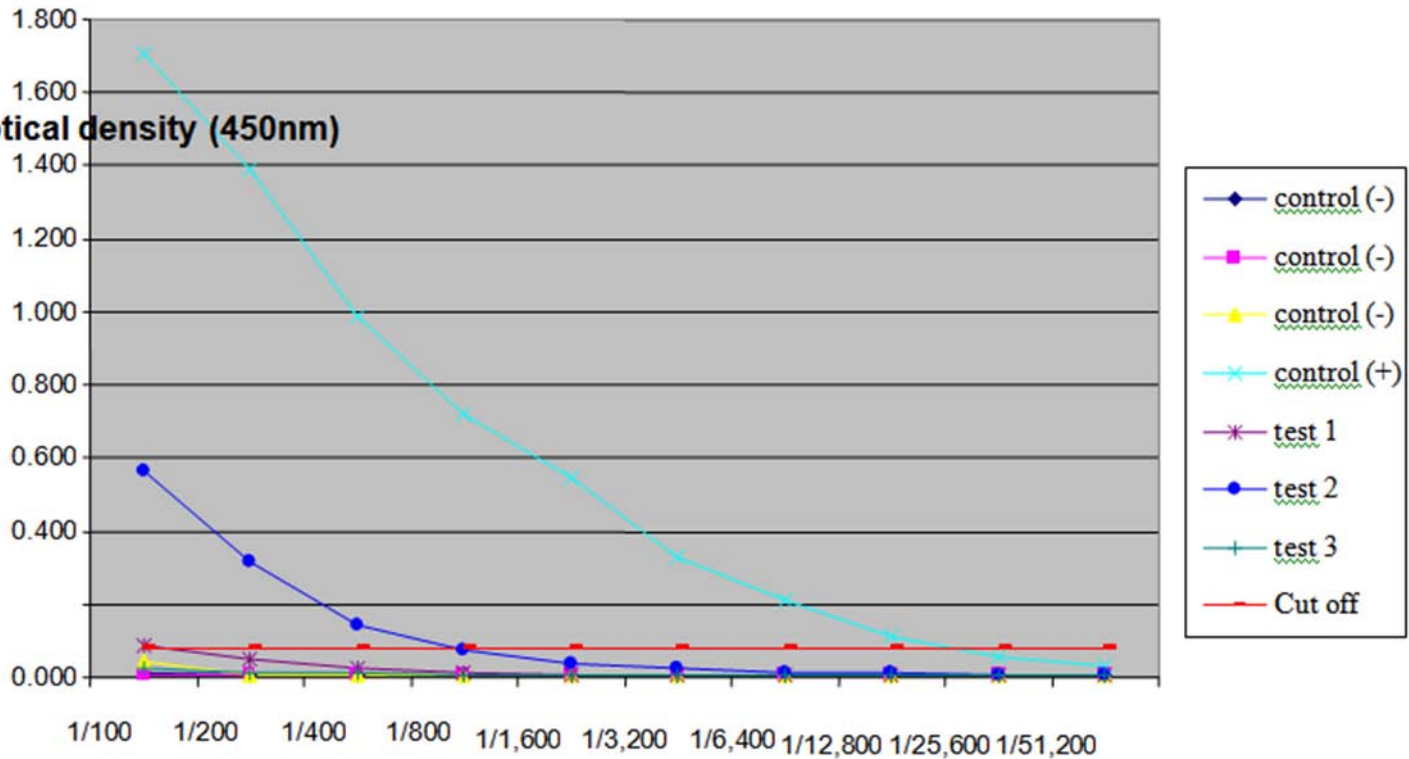

**Sample dilutions**

Supplement: Supplementary file 8 — Authors’ original file for figure 1 [file 13071_2011_563_MOESM8_ESM.pdf]

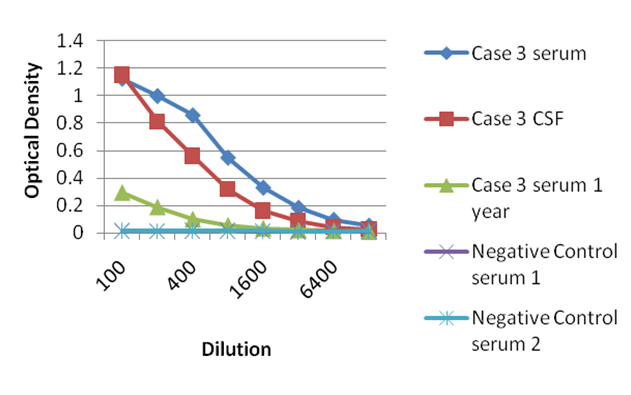

Supplement: Supplementary file 9 — Authors’ original file for figure 2 [file 13071_2011_563_MOESM9_ESM.png]

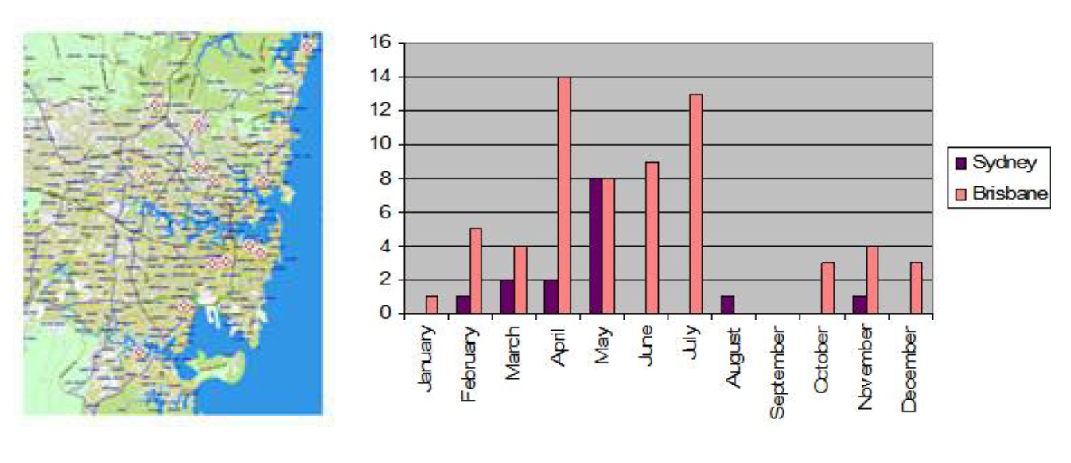

Supplement: Supplementary file 10 — Authors’ original file for figure 3 [file 13071_2011_563_MOESM10_ESM.bmp]

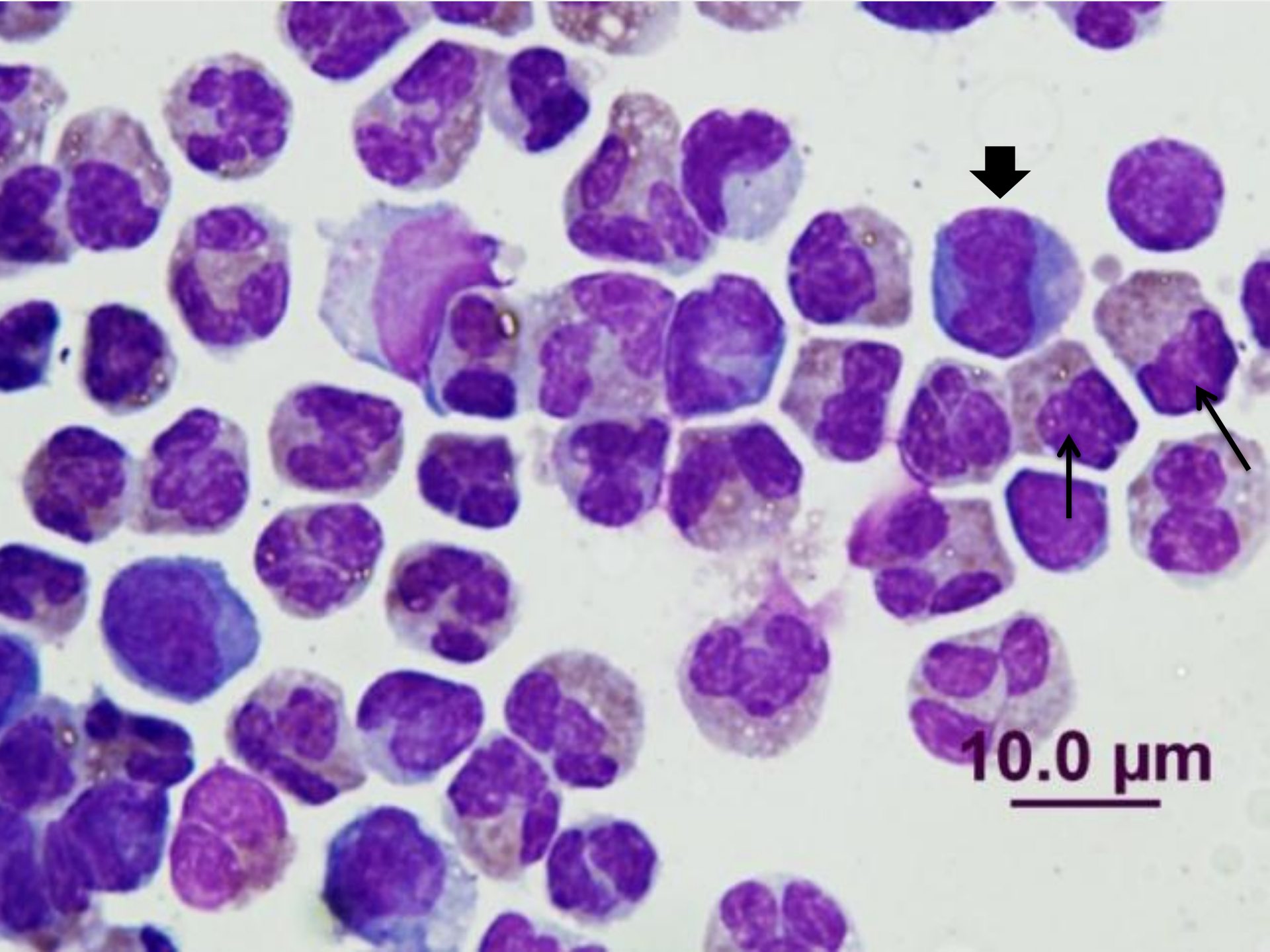

10.0  $\mu\text{m}$

Supplement: Supplementary file 11 — Authors’ original file for figure 4 [file 13071_2011_563_MOESM11_ESM.pdf]

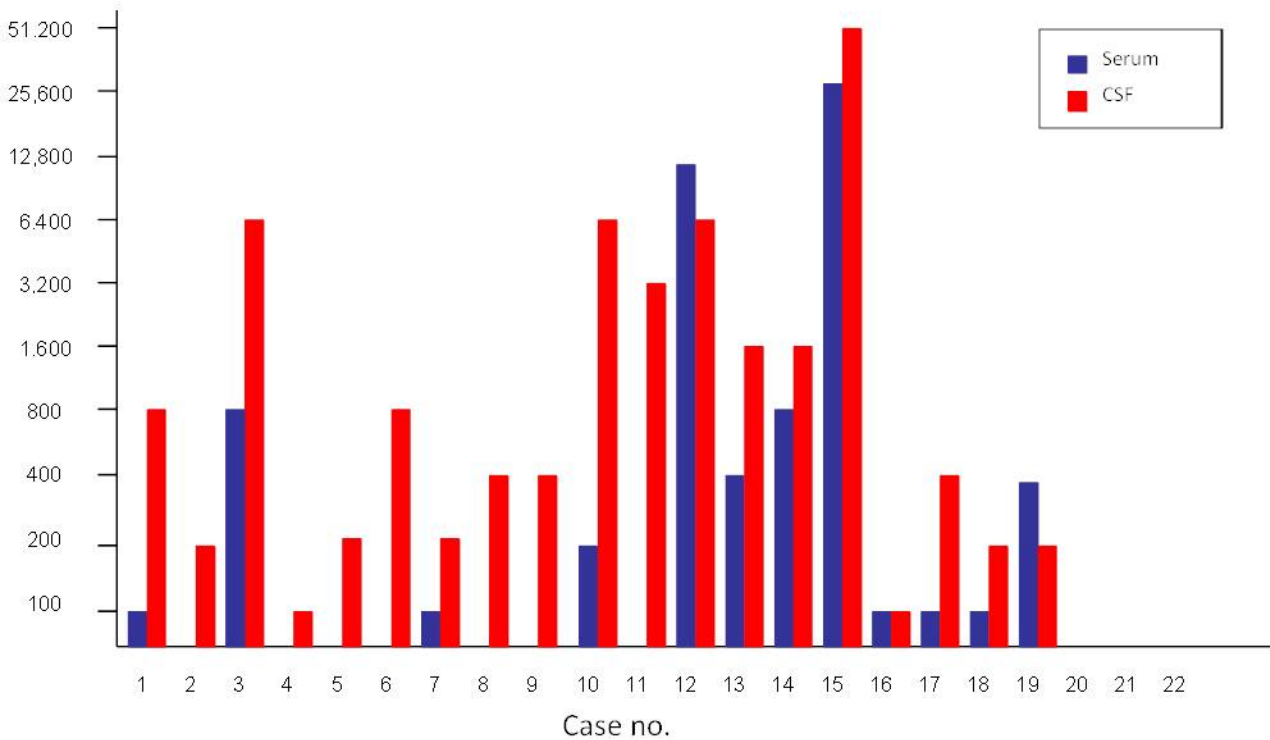

Supplement: Supplementary file 12 — Authors’ original file for figure 5 [file 13071_2011_563_MOESM12_ESM.pdf]

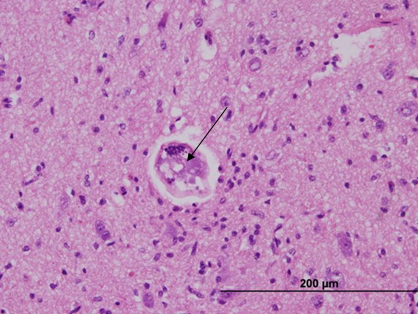

Supplement: Supplementary file 13 — Authors’ original file for figure 6 [file 13071_2011_563_MOESM13_ESM.bmp]

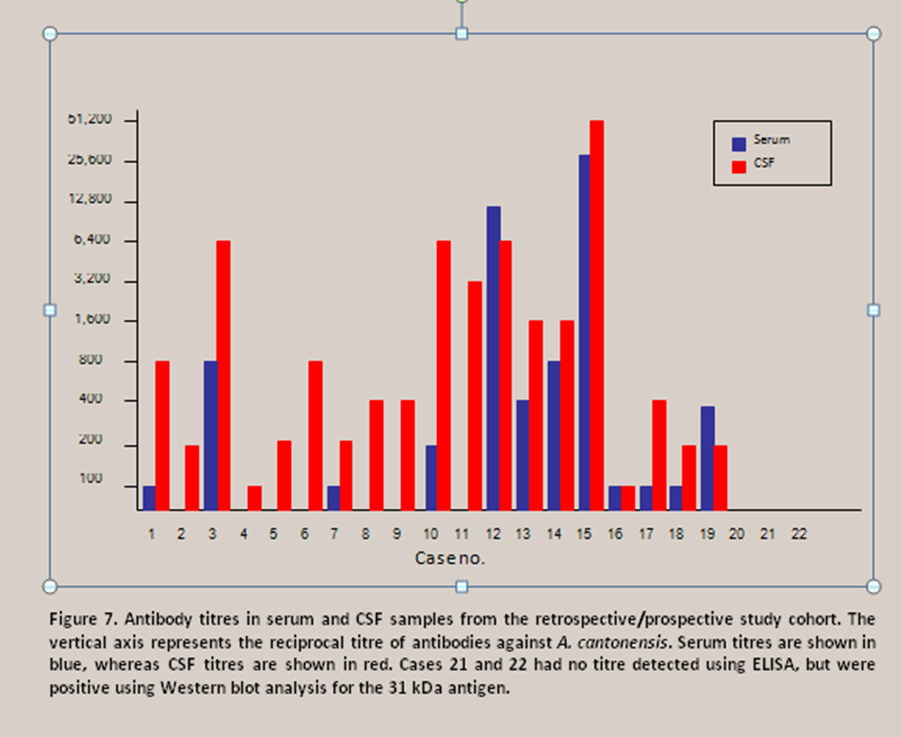

Supplement: Supplementary file 14 — Authors’ original file for figure 7 [file 13071_2011_563_MOESM14_ESM.png]
