# Supplementary material for: Twenty two cases of canine neural angiostrongylosis in eastern Australia (2002-2005) and a review of the literature
Source: Parasit Vectors. 2012 Apr 5;5:70. doi: 10.1186/1756-3305-5-70 (PMC3361490; doi:10.1186/1756-3305-5-70)
Supplement: Supplementary file 2 — Additional file 2: Appendix 2. Questionnaire sent to clinicians with suspect cases of NA. (DOC 66 KB) [file 13071_2011_563_MOESM2_ESM.DOC]

### Appendix 2

**Questionnaire sent to clinicians with suspect cases of NA**

Please confirm the patient details and your contact information:

Patient name:	Species:	Sex: M/F (N)

Age: (y/m)	Breed:

Referring Vet:

Hospital:	Phone:	Fax:

Email:

1. Does the dog have a history of eating slugs or snail? Yes / No

If so what is the relationship between ingestion and the onset of clinical signs?

2. What are the neurological findings? Does the dog have any of the following?

- Hyperaesthesia
- Reduce withdrawal
- Urinary incontinence
- Faecal incontinence
- Lack of proprioception
- Reduced reflexes
- Increased reflexes
- Anal tone
- Ataxia
- Lack of deep pain
- Neck pain

3. Does the dog have normal mentation? If not please describe.

4. Are the neurological signs getting worse? Yes / No

5. Does the dog have any ocular problems? If so please describe.

6. Is the patient on the following medications?

Routine intestinal worm prophylaxis	Yes/No	Which drug?

Heartworm prophylaxis	Yes / No	Which drug?

7. How does this treatment relate to the onset of clinical signs? (Please circle one)

Within one week	Within two weeks	Unrelated

Attention:

Address:

Fax number:

Dear Dr…………..,

Thank you for contacting me regarding you suspected case. There are just a few questions that would help with our research. The sheet can be faxed back to the University Veterinary Centre, Sydney when you have finished. Below is some background information on the disease and the Masters project

### Background: Neural Angiostrongylosis

The most common cause of eosinophilic meningoencephalitis (EME) in humans is neural angiostrongylosis caused by migrating larvae of the rat lungworm *Angiostrongylus cantonensis.* *A. cantonensis* is found throughout the tropical regions of Oceania (South East Asia and Australia) and Central America. In animals species affected include dogs, cats, horses and native species of birds (owls and kingfishers), possums and bats.

Diagnosis of the canine form of neural angiostrongylosis (NA) is presumptive and based on history, characteristic clinical findings, and eosinophilic pleocytosis in the CSF. Typically these patients are puppies that have been known to eat slugs, snails or possibly rats (definitive host), that have ascending CNS dysfunction, in particular hyperaesthesia, and have marked eosinophilic pleocytosis of the CSF. Definitive diagnosis has only been obtained at necropsy. However there is an ELISA that is available in humans which shows promise in definitively diagnosing NA.

Treatment in dogs is confined to immunosuppressive therapy, mainly prednisolone. The pathology of the disease in young animals is thought to be mainly due to the non-definitive host’s inappropriate reaction to the migrating parasitic larvae. It is though that dead or dying larvae produce much more profound inflammation. Consequently the use of anthelmintics in dogs with suspected neural angiostrongylosis has been found, at least in Australia, to worsen the disease. This is not the case in humans where anthelmintics are commonly used to treat eosinophilic meningoencephalitis due to *A. cantonensis*.

# The Project

The main aim of the Masters is to try and improve the ELISA. In Australia the ELISA uses a fairly impure antigen based on the 4^th^ stage larvae of *A. cantonensis*. The ELISA can be run on serum or CSF and certainly in humans it appears that serum is more sensitive. However there is a problem with the test’s specificity when using serum, as there is significant cross-reactivity with other strongyloids. Obviously using serum to test for NA would be preferable so work needs to be done on improving the specificity of the ELISA. Once the ELISA has been established we can then use it to aid the diagnosis in clinical cases.

Stage 1 would be to obtain/make the purified antigens or monoclonal antibodies and then retest all of the samples we have from suspected cases of NA (both from cases at the UVCS and from other clinics). This would tell us whether or not ELISA is a worthwhile tool in the diagnosis of NA. The majority of cases of NA in Australia occur in Queensland so obtaining more samples to validate the ELISA would be beneficial.

## Samples

Serum and CSF are desirable if available. Serial serum samples taken every 2 weeks for 8 weeks after the diagnosis would be extremely helpful! Please note that you can take whole clotted blood and leave it in a fridge overnight before extracting the serum and freezing it if you do not have a centrifuge.

Thank you again for you time in filling in the attached form. Your cooperation is greatly appreciated.

Yours sincerely

Julian Lunn BVSc MACVSc

University Veterinary Centre, Sydney

Evelyn Williams Building B10

Faculty of Veterinary Science

University of Sydney NSW 2006
